# Phytochemical and biotechnological studies on *Schisandra chinensis* cultivar Sadova No. 1—a high utility medicinal plant

**DOI:** 10.1007/s00253-018-8981-x

**Published:** 2018-04-23

**Authors:** Agnieszka Szopa, Marta Klimek-Szczykutowicz, Adam Kokotkiewicz, Anna Maślanka, Agata Król, Maria Luczkiewicz, Halina Ekiert

**Affiliations:** 1 Chair and Department of Pharmaceutical Botany, Faculty of Pharmacy, Medical College, Jagiellonian University, ul. Medyczna 9, 30-688 Kraków, Poland; 2 Chair and Department of Pharmacognosy, Faculty of Pharmacy, Medical University of Gdansk, al. gen. J. Hallera 107, 80-416 Gdańsk, Poland; 3 Department of Inorganic and Analytical Chemistry, Faculty of Pharmacy, Medical College, Jagiellonian University, ul. Medyczna 9, 30-688 Kraków, Poland

**Keywords:** Chinese magnolia vine cultivar, Schisandra lignans, In vitro cultures, Agar culture, Agitated culture, Bioreactor culture, Elicitation

## Abstract

In the presented work, raw materials (fruits and leaves) and in vitro biomass of a highly productive *Schisandra chinensis* Sadova No. 1 cultivar (SchS) were evaluated for the production of therapeutically useful schisandra lignans (SL). In vitro cultures of SchS were initiated, followed by extensive optimization studies focused on maximizing secondary metabolite production, with the aim of establishing a sustainable source of SL. Different cultivation systems (agar, agitated, bioreactor), experiment times (10, 20, 30, 40, 50 and 60 days) and plant growth regulators (6-benzyladenine—BA and 1-naphthaleneacetic acid—NAA, from 0 to 3 mg/l) in Murashige-Skoog (MS) medium were tested. Moreover, an elicitation procedure was applied to bioreactor-grown microshoots in order to increase SL production. Validated HPLC-DAD protocol enabled to detect fourteen SL in the extracts from in vitro and in vivo materials. The main compounds in the in vitro cultures were as follows: schisandrin (max. 176.3 mg/100 g DW), angeloylgomisin Q (max. 85.1 mg/100 g DW), gomisin A (max. 71.4 mg/100 g DW) and angeloylgomisin H (max. 67.0 mg/100 g DW). The highest total SL content (490.3 mg/100 g DW) was obtained in extracts from the biomass of agar cultures cultivated for 30 days on the MS medium variant containing 3 mg/l BA and 1 mg/l NAA. This amount was 1.32 times lower than in fruit extracts (646.0 mg/100 g DW) and 2.04 times higher than in leaf extracts (240.7 mg/100 g DW). The study demonstrated that SchS is a rich source of SL, thus proving its value for medical, cosmetic and food industry.

**Electronic supplementary material:**

The online version of this article (10.1007/s00253-018-8981-x) contains supplementary material, which is available to authorized users.

## Introduction

*Schisandra chinensis* (Turcz.) Baill.—the Chinese magnolia vine, or schisandra (Sch)—is a pharmacopoeial species of documented therapeutic importance (Szopa et al. 2017a). The raw material is the schisandra fruit—*Schisandrae chinensis fructus*; it has monographs not only in the pharmacopoeias of Asian countries (Central Pharmaceutical Affairs Council 2002; Chinese Pharmacopoeia Commission 2005; Committee of the Japanese Pharmacopoeia 2006), but also in the European (European Directorate for the Quality of Medicines 2017) and American Pharmacopoeias (Upton et al. 2011), as well as in the International Pharmacopoeia published by the WHO (World Health Organization 2007). Sch fruits exhibit many valuable biological activities, e.g. hepatoprotective, adaptogenic and ergogenic, antitumour, immunostimulant, anti-inflammatory, anti-ulcer, antioxidant and detoxifying, and also antiviral and antimicrobial. Extracts from the fruit have a positive effect on the functioning of the cardiovascular and respiratory systems, and show anti-osteoporotic and anti-obesity activities (Hancke et al. 1999; Szopa et al. 2017a). They are also used in cosmetic industry as antioxidant, immunostimulant and antiphlogistic agent (Szopa et al. 2016a). The valuable pharmacological and cosmetological properties are determined by the unique chemical composition of Sch. The main group of secondary metabolites which are unique to this species and responsible for its biological activities is dibenzocyclooctadiene lignans, also called ‘schisandra lignans’ (SL) (Opletal et al. 2004).

Hitherto, we have analysed SL in fruit and leaf extracts from Sch (Szopa and Ekiert 2011). Moreover, different types of Sch in vitro cultures (agar, agitated, stationary liquid and bioreactor-grown), were analysed which can be considered as a potential alternative source of aforementioned compounds (Szopa et al. 2016b, 2017b, 2018).

In the present study, a cultivated variety of *S*. *chinensis*, namely *S. chinensis* cv. Sadova No. 1 (further referred to as ‘SchS’), was investigated for SL production. SchS is a Ukrainian cultivar of Sch, which was selected by Ivan Shaitan in the M. M. Grishko National Botanical Garden in Kiev. The SchS cultivar entered the State Register of Plants in Ukraine in 1998 (Shaitan 2005). It was created from selected seeds of wild Sch specimens growing in Primorsk. The resulting plants are characterized by vigorously growing shoots and large clusters of fruits. The fruits have a sour taste, juicy flesh and bright red juice. As compared to original species, the Sadova cultivar is more productive and resistant to harsh climate events (Shaitan 2005). However, according to the data revealed by literature review, little is known about the chemical composition of SchS. The fruits of SchS were shown to contain ascorbic acid, sugars and organic acids (Shaitan 2005), but there are no data on the levels of the main group of schisandra secondary metabolites, i.e. SL. Given the above, aims of the current study were formulated: firstly, to evaluate SL content in leaves and fruits of SchS, following to establish in vitro cultures of SchS for sustainable production of SL.

The biotechnological experiments (upstream procedures) included culture initiation and optimization of the growth conditions in terms of selecting the culture type (agar, agitated, bioreactor—Plantform temporary immersion system), culture duration (10, 20, 30 40, 50, 60 days) and plant growth regulators (PGRs) used (BA and NAA; in the concentration range from 0 to 3 mg/l) in Murashige and Skoog medium (MS, (Murashige and Skoog 1962)). Moreover, in order to increase the production of SL, the elicitation strategy with yeast extract was performed on microshoots cultivated in a Plantform bioreactor.

The high-performance liquid chromatography with diode array detection (HPLC-DAD) was selected for all phytochemical analyses (downstream procedures) which included validation of the method as far as determination of SL was concerned.

To the best of our knowledge, this is the first report describing a phytochemical analysis of SL in fruit and leaf extracts of SchS. Moreover, innovative biotechnological approach was employed in order to establish sustainable production of SL.

## Materials and methods

### Origin of plant material

The plant material for the study was a specimen of the parent plant—*Schisandra chinensis* Sadova No. 1 (SchS) growing in a commercial nursery, Clematis—Źródło Dobrych Pnączy Spółka z o.o.—is a limited partnership company with its registered office in Pruszków (ul. Duchnicka 27, 05-800 Pruszków, Poland) (http://www.clematis.com.pl/). The plant was taxonomically verified by the scientific staff of the Clematis arboretum. The plant material consisted of the leaves and fruits of SchS harvested in August 2015. After harvesting, the leaves and fruits were preserved by lyophilization (Labconco lyophilizer).

### In vitro cultures

#### Initiation of in vitro cultures

The material for establishment of in vitro cultures consisted of apical buds (obtained in spring 2015) from the SchS specimen described above (section ‘Origin of plant material’). The plant material was treated with 70% ethanol (30 s); then, it was disinfected with a 0.1% HgCl_2_ solution for 10 min, washed three times with sterile water and inoculated onto the standard MS (Murashige and Skoog 1962) medium with 0.8% (*w/v*) agar (plant agar, Duchefa), 3% (*w/v*) sucrose, 1 mg/l BA (6-benzyladenine) and 0.5 mg/l NAA (1-naphthaleneacetic acid). After approx. 8-weeks, viable, green microshoot cultures were established. The in vitro cultures were maintained at 25 ± 2 °C under continuous artificial illumination at 2.75 W/m^2^ (LF-40 W lamp, daylight, Piła) and subcultured at 4-week intervals.

#### Experimental in vitro cultures

##### Agar microshoot cultures

**Fig. 1:**
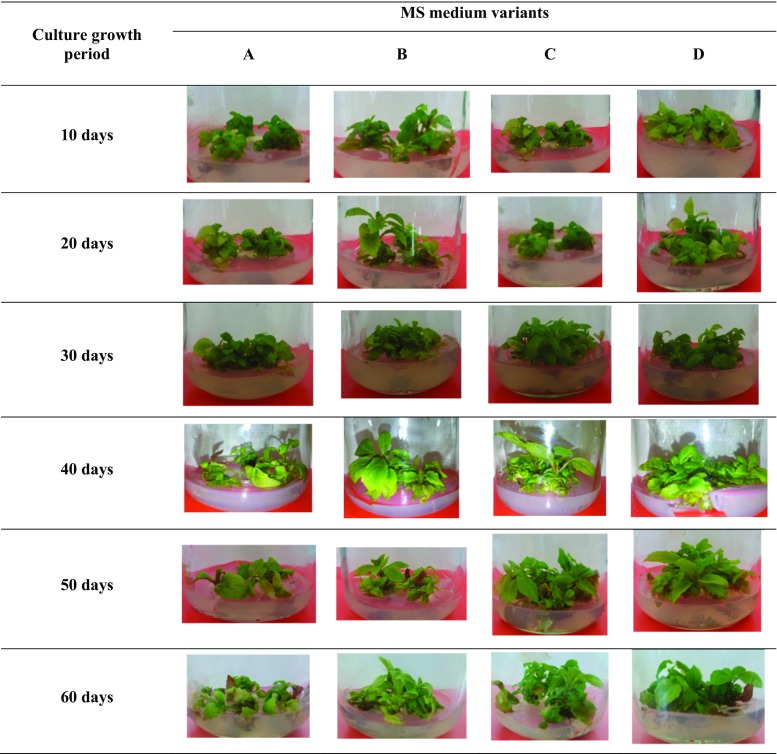

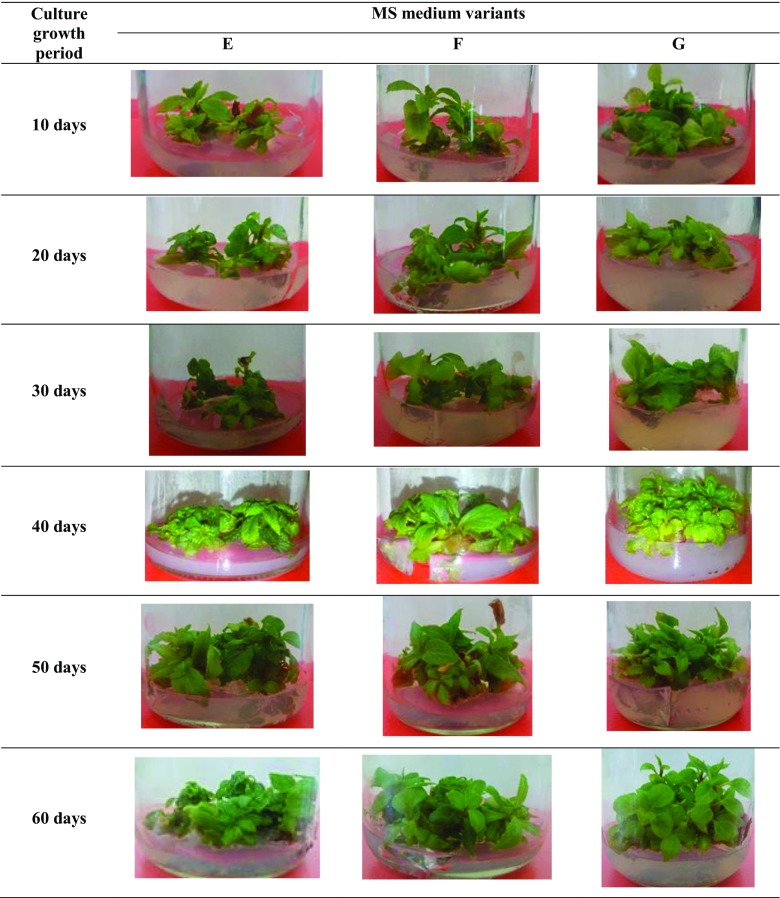
Morphological appearance of *S*. *chinensis* cv. Sadova microshoots grown for 10–60 days on different MS (Murashige and Skoog) agar media variants: A—control (without PGRs), B—0.1 mg/l BA and 2 mg/l NAA, C—0.5 mg/l BA and 2 mg/l NAA, D—2 mg/l BA and 0.5 mg/l NAA, E—2 mg/l BA and 1 mg/l NAA, F—2 mg/l BA and 2 mg/l NAA, G—3 mg/l BA and 1 mg/l NAA

The study involved cultivating microshoots of SchS. Two types of cultures were tested: stationary (agar) and agitated (liquid). In the stationary system, shoot cultures (Fig. 1; Online resource) were maintained on different variants of the standard MS (Murashige and Skoog 1962) medium with 0.8% (w/v) agar (plant agar, Duchefa) and 3% (*w/v*) sucrose. The tested media differed with respect to the concentrations of plant growth regulators (PGRs)—BA and NAA (mg/l): 0.1 and 2 (variant B), 0.5 and 2 (variant C), 2 and 0.5 (variant D), 2 and 1 (variant E), 2 and 2 (variant F) and 3 and 1 (variant G). The medium combination without PGRs (control) was designated as variant A. The inoculum was 0.5 g of shoots, taken from the previously established microshoot culture (section ‘Initiation of in vitro cultures’) on the 30^th^ day of the growth cycle. The experimental cultures were maintained in Magenta™ vessels (Sigma, product No. V8630) containing 30 ml of MS medium. Microshoots were grown under continuous white light (2.75 W/m^2^), at 25 ± 2 °C, over 60-day growth periods. The biomass was collected at 10-day intervals, after 10, 20, 30, 40, 50 and 60 days of the experiment.

##### Agitated microshoot cultures

**Fig. 2:**
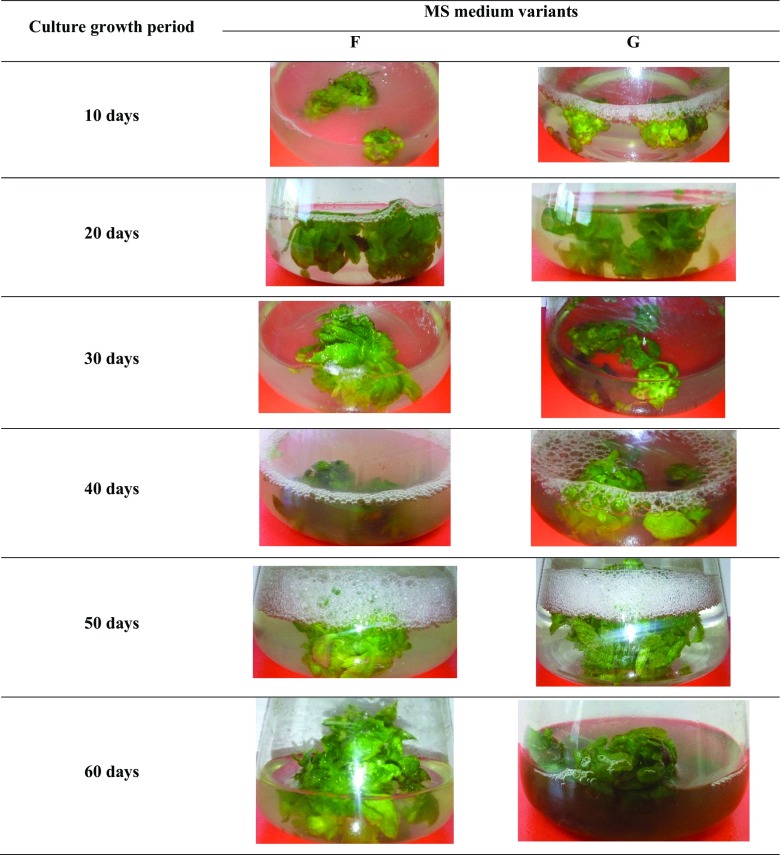
Morphological appearance of *S. chinensis* cv. Sadova agitated microshoots grown for 10–60 days in different liquid media variants: F—2 mg/l BA and 2 mg/l NAA, G—3 mg/l BA and 1 mg/l NAA

Agitated microshoot cultures (Fig. 2) were cultivated in 300 ml Erlenmeyer flasks containing 100 ml of MS medium, on a rotary shaker (Altel) at 140 rpm. The inoculum was composed of 1.5 g microshoot sections (prepared as described in ‘Agar microshoot cultures’). The agitated cultures were maintained on two variants (F and G, as described in ‘Agar microshoot cultures’) of the standard liquid MS (Murashige and Skoog 1962) medium with 3% (*w/v*) sucrose and containing respectively: 2 mg/l BA and 2 mg/l NAA—variant F, and 3 mg/l BA and 1 mg/l NAA—variant G (the choice of growth regulator compositions was made due to the fact that these variants had the best growth and production results for agar cultures).

The nutrient media and biomass samples from the agitated cultures were collected at 10-day intervals on 10, 20, 30, 40, 50 and 60 day of the culture. SchS microshoots were grown under continuous white light (2.75 W/m^2^), at of 25 ± 2 °C.

##### Bioreactor cultures

**Fig. 3:**
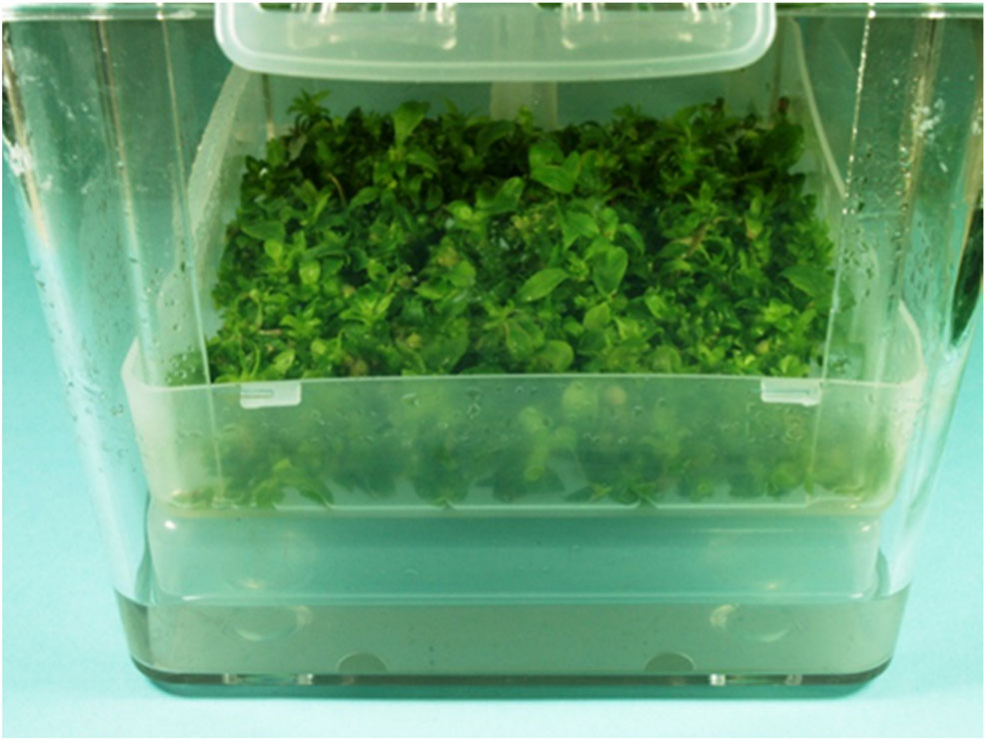
*S. chinensis* cv. Sadova microshoots grown for 30 days in temporary immersion system (Plantform bioreactor) on MS medium variant G (3 mg/l BA and 1 mg/l NAA)

SchS shoots were grown for 30 days in the Plantform temporary immersion system (Plant Form, Sweden) (Fig. 3), following the protocol described previously (Szopa et al. 2017b). The bioreactor was inoculated at 15/500 (g/ml) microshoots to medium ratio (variant G of MS medium of 3 mg/l BA and 1 mg/l NAA) (the best growth and production medium for SchS microshoots). The immersion cycle was set to 5 min every 1.5 h, at 1.0 vvm aeration rate.

For the Plantform bioreactor experiments, an elicitation with 1000 mg/l of yeast extract (YE) (plant cell culture tested, Sigma-Aldrich), added on day 20 of the 30-day growth period, was applied, according to elicitation protocol described previously (Szopa et al. 2018). The YE for the elicitor treatments was steam-sterilized (120 °C, 20 min, 1 bar) prior to use. For elicitation, one of the side hose nipples of the Plantform bioreactor was used as an inlet port. Samples of the microshoots and the media were collected on day 30 of the experiment.

### Chromatographic assays

#### Extraction

For the preparation of methanolic extracts, 0.3 g portions of lyophilized (Labconco lyophilizer) and pulverized matrices (intact plant material and biomass from in vitro cultures) were weighed out. The material was subjected to extraction with 5-ml methanol (STANLAB, Poland) by sonication in an ultrasonic bath (POLSONIC 2, Poland) for 30 min. Freeze-dried samples of the experimental media from agitated and bioreactor cultures were also subjected to extraction in this manner. The obtained extracts were filtered through sterilizing syringe filters (0.22 μm, Millex^®^GP, Millipore) prior to HPLC analysis.

#### Phytochemical analysis

**Fig. 4:**
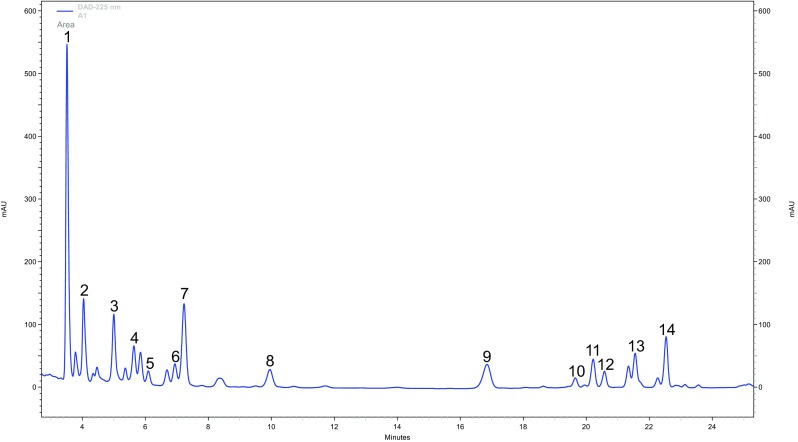
The representative HPLC-UV chromatogram (*λ* = 225 nm) of a methanol extract of *S. chinensis* cv. Sadova biomass cultivated in vitro. SL: 1—schisandrin, 2—gomisin, 3—angeloyl-/tigloylgomisin, 4—angeloyl-/tigloylgomisin Q, 5—gomisin G, 6—schisantherin A, 7—schisantherin B, 8—schisanthenol, 9—deoxyschisandrin, 10—schisandrin B, 11—*γ*-schisandrin, 12—benzoylgomisin P, 13—schisandrin, 14—schisantherin D

Analysis of dibenzocyclooctadiene lignans (SL) was performed by HPLC-DAD according to the method originally developed by Zhang et al. (Zhang et al. 2009) for Sch fruits, and subsequently adapted for Sch in vitro cultures in the course of our previous work (Szopa et al. 2016b). The Hitachi LaChrom Elite^®^ HPLC system was used equipped with a DAD detector L-2455, column oven L-2350 and autosampler L-2200r. Separation was performed using a Kinetex™ C-18 analytical column (150 × 4.6 mm, 2.6-μm particle size; Phenomenex, USA) at 30 °C. The mobile phase consisted of acetonitrile (A) and water (B), with a flow-rate of 0.8 ml/min. The gradient program was as follows: 0–4 min, 40–45; 4–12 min, 45–50; 12–16 min, 50–68; 16–20 min, 68–75; 20–25 min, 75–95 (% B), with a hold time of 15 min; injection volume was 5 μl. Detection wavelength was set at 225 nm. Identification and quantification were made by comparison of UV spectra and retention times with nine standards: gomisin A, deoxyschisandrin, schisandrin and *γ*-schisandrin (ChromaDex^®^, USA), gomisin G and schisantherin A (PhytoLab GmbH & Co. KG, Germany), schisandrin C, schisantherin B and schisanthenol (ChemFaces Biochemical Co. Ltd., China). Additionally, based on our previous studies (Szopa et al. 2016b), quantification of five tentatively identified compounds, schisandrin B, benzoylgomisin P, angeloyl-/tigloylgomisin H, angeloyl-/tigloylgomisin Q, schisantherin D (or one of its stereoisomers—benzoylgomisin O or benzoylisogomisin O) (based on HPLC-DAD-ESI/MS analyses), was performed using the calibration curve for schisandrin, as the main component of the group of SL. The representative HPLC-UV chromatogram (*λ* = 225 nm) of a methanol extract of SchS biomass cultivated in vitro was presented in Fig. 4.

#### Validation

Validation of the HPLC-DAD method was performed by determination of accuracy, precision, linearity, limit of detection and limit of quantification (European Medicines Agency 2005).

#### Accuracy

Accuracy was determined based on sample analysis of known concentrations and comparing the results obtained by a validated method with true values, followed by calculation of the recovery percentage. Determinations were performed at three concentration levels: 80, 100 and 120%, for each of them three repetitions were done.

#### Precision

Precision of the method was determined at three levels of substance concentrations in reference solutions: 50, 100 and 150%. For each level of concentration, three repetitions were done.

#### Linearity

Linearity was determined by comparing the relationship between peak area and a concentration of the tested substances (mg/ml). Two series of assays in the following concentration range were made for deoxyschisandrin: from 0.0039 to 1.0000 mg/ml, for *γ*-schisandrin: from 0.0013 to 1.0000 mg/ml and in the following concentration ranges: 0.0200 to 0.2000 mg/ml for other SL.

#### Limit of detection and limit of quantification

The limit of detection (LOD) and limit of quantification (LOQ) were determined from the linearity in the following concentration ranges: from 0.0039 to 0.0312 mg/ml for deoxyschisandrin, from 0.0013 to 0.0050 mg/ml for *γ-*schisandrin and from 0.0200 to 0.1000 mg/ml for other SL, using the following formulas: LOD = 3.3 × *S*_y_/*a*, LOQ = 10 × *S*_y_/*a*, where *S*_y_ is the estimation error and *a* is the slope.

### Statistical analysis

The data presented, were organized with independent samples (three replications). Analyses and measurements of each sample were performed in triplicate and average values were used for statistical evaluation. The significant differences in the individual and total contents of estimated SL contents were compared with the one-way analysis of variance (one-way ANOVA). For comparison and contrast between different amounts, post hoc Tukey HSD (honestly significant difference) test was used. Outcomes demonstrating *p* < 0.05 were considered statistically significant. Data analysis was performed using STATISTICA 13 PL (StatSoft) software.

The experiments have been repeated thrice. The results were presented as mean ± standard deviation (SD). The STATISTICA version 13 PL software package (StatSoft) was used for the analysis.

## Results

### Validation setup

The validation results presented in Table 1 indicate that the proposed HPLC-DAD method is characterized by high sensitivity; LOD for deoxyschisandrin is 0.0089 mg/ml, 0.0050 mg/ml for schisandrin, 0.0120 mg/ml for gomisin A, 0.0048 mg/ml for *γ-*schisandrin, 0.0044 mg/ml for schisandrin C, 0.0095 mg/ml for schisanthenol, 0.0151 mg/ml for schisantherin B, 0.0103 mg/ml for gomisin G and 0.0093 mg/ml for schisantherin A. LOQ was estimated at 0.0269, 0.0151, 0.0372, 0.0145, 0.0133, 0.0288, 0.0457, 0.0312, 0.0185 and 0.0282 mg/ml, respectively. Percentage recovery of the studied compounds presented as mean values for three concentration levels is high and ranges from 95.77 to 103.90%. Satisfactory precision determined for three concentration levels is confirmed by the values of variability coefficients RSD which are in the range from 0.21 to 2.96%. Linearity of the tested substances was preserved in a wide range: from 0.0039 to 1.0000 mg/ml for deoxyschisandrin, from 0.0013 to 1.0000 mg/ml for *γ*-schisandrin and in the following concentration ranges: 0.0200 to 0.2000 mg/ml for other substances.

**Table 1:** Validation of the developed methods with statistical evaluation for HPLC method

Validation parameters	Schisandrin	Gomisin A	Gomisin G	Schisantherin A	Schisantherin B	Schisanthenol	Deoxy-schisandrin	*γ-*Schisandrin	Schisandrin C
*t*_R_ [min]	3.373	3.833	5.693	6.073	7.133	7.533	16.273	20.093	20.293
LOD [mg/ml]	0.0050 *S*_y_ = 159,600 *a* = 105,634,056	0.0120 *S*_y_ = 581,800 *a* = 156,478,588	0.0103 *S*_y_ = 554,300 *a* = 177,487,349	0.0093 *S*_y_ = 211,000 *a* = 14,907,025	0.0151 *S*_y_ = 550,000 *a* = 120,405,034	0.0095 *S*_y_ = 448,300 *a* = 155,693,204	0.0089 *S*_y_ = 541,700 *a* = 201,407,288	0.0048 *S*_y_ = 451,300 *a* = 309,168,100	0.0044 *S*_y_ = 195,200 *a* = 147,188,677
LOQ [mg/ml]	0.0151	0.0372	0.0312	0.0282	0.0457	0.0288	0.0269	0.0145	0.0133
Recovery 80% [%]	$\overline{x}$ = 98.70 *S*_x_ = 0.721 RSD = 0.73%	$\overline{x}$ = 101.77 *S*_x_ = 0.416 RSD = 0.41%	$\overline{x}$ = 96.17 *S*_x_ = 0.666 RSD = 0.69%	$\overline{x}$ = 103.60 *S*_x_ = 0.656 RSD = 0.63%	$\overline{x}$ = 100.30 *S*_x_ = 0.656 RSD = 0.65%	$\overline{x}$ = 10,197 *S*_x_ = 0.351 RSD = 0.34%	$\overline{x}$ = 96.50 *S*_x_ = 0.458 RSD = 0.47%	$\overline{x}$ = 98.97 *S*_x_ = 0.702 RSD = 0.71%	$\overline{x}$ = 101.77 *S*_x_ = 0.306 RSD = 0.30%
Recovery 100% [%]	$\overline{x}$ = 102.93 *S*_x_ = 0.305 RSD = 0.30%	$\overline{x}$ = 101.73 *S*_x_ = 0.208 RSD = 0.20%	$\overline{x}$ = 95.77 *S*_x_ = 0.451 RSD = 0.47%	$\overline{x}$ = 102.10 *S*_x_ = 0.781 RSD = 0.76%	$\overline{x}$ = 101.60 *S*_x_ = 0.400 RSD = 0.39%	$\overline{x}$ = 101.63 *S*_x_ = 0.577 RSD = 0.57%	$\overline{x}$ = 103.07 *S*_x_ = 0.751 RSD = 0.73%	$\overline{x}$ = 102.90 *S*_x_ = 0.520 RSD = 0.50%	$\overline{x}$ = 101.90 *S*_x_ = 0.400 RSD = 0.39%
Recovery 120% [%]	$\overline{x}$ = 98.60 *S*_x_ = 0.794 RSD = 0.80%	$\overline{x}$ = 99.33 *S*_x_ = 0.666 RSD = 0.67%	$\overline{x}$ = 99.43 *S*_x_ = 1.050 RSD = 1.06%	$\overline{x}$ = 103.57 *S*_x_ = 0.404 RSD = 0.39%	$\overline{x}$ = 101.10 *S*_x_ = 0.500 RSD = 0.49%	$\overline{x}$ = 98.67 *S*_x_ = 0.289 RSD = 0.29%	$\overline{x}$ = 103.00 *S*_x_ = 1.389 RSD = 1.35%	$\overline{x}$ = 101.97 *S*_x_ = 0.351 RSD = 0.34%	$\overline{x}$ = 99.00 *S*_x_ = 0.624 RSD = 0.63%
Precision 50% c [mg/ml]	$\overline{x}$ = 0.0513 *S*_x_ = 0.00059 RSD = 1.14%	$\overline{x}$ = 0.0507 *S*_x_ = 0.00058 RSD = 1.14%	$\overline{x}$ = 0.0585 *S*_x_ = 0.00026 RSD = 0.45%	$\overline{x}$ = 0.0605 *S*_x_ = 0.00117 RSD = 1.93%	$\overline{x}$ = 0.0509 *S*_x_ = 0.00016 RSD = 0.32%	$\overline{x}$ = 0.0503 *S*_x_ = 0.000675 RSD = 1.34%	$\overline{x}$ = 0.0509 *S*_x_ = 0.00026 RSD = 0.52%	$\overline{x}$ = 0.0618 *S*_x_ = 0.00045 RSD = 0.73%	$\overline{x}$ = 0.0493 *S*_x_ = 0.00055 RSD = 1.11%
Precision 100% c [mg/ml]	$\overline{x}$ = 0.1004 *S*_x_ = 0.00051 RSD = 0.51%	$\overline{x}$ = 0.1121 *S*_x_ = 0.00332 RSD = 2.96%	$\overline{x}$ = 0.1181 *S*_x_ = 0.00185 RSD = 1.57%	$\overline{x}$ = 0.1002 *S*_x_ = 0.00038 RSD = 0.19%	$\overline{x}$ = 0.1075 *S*_x_ = 0.00134 RSD = 1.25%	$\overline{x}$ = 0.1101 *S*_x_ = 0.00044 RSD = 0.40%	$\overline{x}$ = 0.1080 *S*_x_ = 0.00195 RSD = 1.80%	$\overline{x}$ = 0.1286 *S*_x_ = 0.00067 RSD = 0.52%	$\overline{x}$ = 0.1043 *S*_x_ = 0.00176 RSD = 1.68%
Precision 150% c [mg/ml]	$\overline{x}$ = 0.1491 *S*_x_ = 0.00055 RSD = 0.37%	$\overline{x}$ = 0.1503 *S*_x_ = 0.00051 RSD = 0.34%	$\overline{x}$ = 0.1588 *S*_x_ = 0.00112 RSD = 0.71%	$\overline{x}$ = 0.1491 *S*_x_ = 0.00342 RSD = 2.30%	$\overline{x}$ = 0.1500 *S*_x_ = 0.00032 RSD = 0.21%	$\overline{x}$ = 0.1492 *S*_x_ = 0.00056 RSD = 0.37%	$\overline{x}$ = 0.1472 *S*_x_ = 0.00407 RSD = 2.76%	$\overline{x}$ = 0.1562 *S*_x_ = 0.00067 RSD = 0.34%	$\overline{x}$ = 0.1489 *S*_x_ = 0.00069 RSD = 0.46%
Linearity	*p* = 89,385,107⋅c+244,667 *r* = 0.99123	*p* = 157,638,344⋅c−321,533 *r* = 0.99855	*p* = 177,427,247⋅c−261,938 *r* = 0.99499	*p* = 66,683,463⋅c+909,038 *r* = 0.99801	*p* = 131,735,836⋅c−189,436 *r* = 0.99794	*p* = 192,658,623⋅c−1,571,419 *r* = 0.99418	*p* = 125,167,085⋅c+1,309,458 *r* = 0.99910	*p* = 317,038,545⋅c+4,655,729 *r* = 0.99851	*p* = 156,110,000⋅c−591,466 *r* = 0.999592

*t*_*R*_ retention time, $\overline{x}$ mean value, *S*_*x*_ standard deviation, *RSD* relative standard deviation, *c* concentration [mg/ml], *S*_*y*_ standard error of the estimate, *a* the slope of regression line

### Phytochemical analysis of plant material

#### Fruits

The SchS fruit extracts were found to contain all of the 14 lignans analysed. The concentrations of the individual compounds varied within a broad range from 2.6 to 166.8 mg/100 g DW (dry weight). In quantitative terms, the dominant metabolites were as follows: schisandrin (166.8 mg/100 g DW), *γ*-schisandrin (96.2 mg/100 g DW), gomisin A (72.4 mg/100 g DW), angeloylgomisin H (71.6 mg/100 g DW) and schisantherin B (56.8 mg/100 g DW). The total SL content in fruit extracts was 646.0 mg/100 g DW (Table 2).

**Table 2:** Schisandra lignans contents (mg/100 g DW±SD) in fruit and leaves extracts of *S. chinensis* cv. Sadova and *S. chinensis*

Lignans	*S. chinensis* cv. Sadova		S. chinensis*	
	Fruits	Leaves	Fruits	Leaves
Schisandrin	166.8 ± 11.0^bcd^	55.1 ± 3.6^acd^	132.4 ± 9.4^abd^	29.7 ± 1.4^abc^
Gomisin A	72.4 ± 12.2^bcd^	12.5 ± 3.7^acd^	109.4 ± 8.3^abd^	34.5 ± 2.2^abc^
Angeloyl-/tigloylgomisin H	71.6 ± 8.3^bcd^	31.4 ± 1.5^acd^	161.9 ± 10.1^abd^	51.2 ± 5.8^abc^
Angeloyl-/tigloylgomisin Q	24.5 ± 6.3^bcd^	13.4 ± 0.4^acd^	52.3 ± 4.9^abd^	8.2 ± 1.1^abc^
Gomisin G	4.8 ± 0.4^bcd^	1.9 ± 0.3^acd^	46.1 ± 3.1^ab^	49.1 ± 2.9^ab^
Schisantherin A	19.5 ± 2.3^bcd^	3.5 ± 0.2^acd^	25.5 ± 2.5^ab^	25.9 ± 3.1^ab^
Schisantherin B	56.8 ± 8.2^bcd^	4.6 ± 0.5^acd^	4.7 ± 1.0^ad^	3.4 ± 0.3^abc^
Schisanthenol	2.6 ± 0.2^bcd^	1.4 ± 0.2^acd^	3.6 ± 0.4^abd^	2.7 ± 0.5^bc^
Deoxyschisandrin	31.2 ± 2.5^bcd^	16.1 ± 2.7^acd^	60.7 ± 5.2^abd^	41.1 ± 3.8^abc^
Schisandrin B	58.9 ± 3.5^bd^	11.9 ± 2.1^acd^	56.8 ± 3.1^bc^	21.8 ± 1.3^abc^
γ-Schisandrin	96.2 ± 4.0^bcd^	24.5 ± 1.7^ac^	66.5 ± 2.5^abd^	22.3 ± 2.0^ac^
Benzoylgomisin P	12.4 ± 1.9^bd^	23.0 ± 2.8^acd^	13.4 ± 0.4^ab^	14.4 ± 1.1^ab^
Schisandrin C	7.0 ± 0.2^bd^	10.1 ± 0.3^ac^	6.1 ± 1.1^abd^	10.9 ± 1.3^ac^
Schisantherin D	21.3 ± 2.3^bcd^	31.3 ± 2.3^acd^	15.2 ± 0.9^abd^	7.8 ± 0.4^abc^
Total content	646.0 ± 63.3^bcd^	240.7 ± 22.3^acd^	754.6 ± 52.8^abd^	322.8 ± 27.1^abc^

*According to Szopa et al. 2016b

^a^*p* < 0.05 vs. *S. chinensis* cv. Sadova Fruits

^b^*p* < 0.05 vs. *S. chinensis* cv. Sadova Leaves

^c^*p* < 0.05 vs. *S. chinensis* Fruits

^d^*p* < 0.05 vs. *S. chinensis* Leaves

#### Leaves

The SchS leaf extracts were found to contain all of the 14 lignans analysed. The concentrations of the individual compounds varied within a broad range from 1.4 to 55.1 mg/100 g DW. The main metabolites were as follows: schisandrin (55.1 mg/100 g DW), angeloylgomisin H (31.4 mg/100 g DW), schisantherin D (31.3 mg/100 g DW) and *γ*-schisandrin (24.5 mg/100 g DW). The total SL content in leaf extracts was 240.7 mg/100 g DW (Table 2).

### Phytochemical analysis of in vitro cultures

#### Agar microshoot cultures

Microshoots growing on the six agar variants of MS medium, containing different concentrations of PGRs, especially on variants F and G, were characterized by a large number of microshoots and a dark green color of the leaves. The shoots growing on variant A of MS medium (control without PGRs) had a smaller number of microshoots and a pale green colour (Fig. 1). The resulting values of the growth index (Gi) ranged from 2.3 to 8.7, depending on the medium variant and culture duration. The highest values were obtained for microshoots cultivated for 60 days on variant G of the medium (Table 3).

**Table 3:** Changes in growth index–Gi (calculated according to the formula: Gi = [(Dw1 − Dw0)/Dw0] where Dw1 is the dry weight of microshoots at the end of experiment and Dw0 is the dry weight of the inoculum), of *S. chinensis* cv. Sadova biomass from all tested in vitro systems (agar, agitated and Plantform bioreactor-grown cultures). Values represent the mean ± SD of four samples

Type of *S. chinensis* cv. Sadova microshoot cultures	MS medium variant*	Culture growth period					
		10 days	20 days	30 days	40 days	50 days	60 days
Agar	A	2.7 ± 0.1^cdef^	2.9 ± 0.2^cdef^	3.5 ± 0.1^abef^	3.5 ± 0.4^abef^	3.8 ± 0.3^abcdf^	3.9 ± 0.6^abcde^
	B	2.5 ± 0.1^bcdef^	3.8 ± 0.3a^cdef^	4.4 ± 0.2^abdef^	4.0 ± 0.6^abcef^	5.2 ± 0.5^abcdf^	5.7 ± 0.3^abcde^
	C	2.5 ± 0.3^bcdef^	4.1 ± 0.2^acdef^	3.7 ± 0.5^abef^	3.7 ± 0.2^abef^	7.2 ± 0.2^abcdf^	5.7 ± 0.3^abcde^
	D	2.5 ± 0.1^bcdef^	3.5 ± 0.5^adef^	3.5 ± 0.2^adef^	4.1 ± 0.5^abcef^	4.9 ± 0.1^abcdf^	6.3 ± 0.8^abcde^
	E	2.3 ± 0.2^bcdef^	2.8 ± 0.2^acdef^	3.1 ± 0.3^abdef^	5.0 ± 0.6^abced^	6.1 ± 0.1^abcdf^	5.8 ± 0.3^abcde^
	F	2.5 ± 0.1^bcdef^	3.2 ± 0.6^acdef^	3.6 ± 0.3^abdef^	5.6 ± 0.8^abcef^	5.3 ± 0.2^abcdf^	6.8 ± 0.7^abcde^
	G	2.8 ± 0.1^bcdef^	3.4 ± 0.5^acdef^	4.8 ± 0.6^abdef^	6.8 ± 0.2^abcef^	7.1 ± 0.1^abcdf^	8.7 ± 0.6^abcde^
Agitated	F	1.9 ± 0.1^bcdef^	2.3 ± 0.2^acdf^	2.9 ± 0.4^abdef^	3.4 ± 0.5^abcef^	2.3 ± 0.1^acdf^	7.5 ± 0.4^abcde^
	G	1.4 ± 0.1^bcdef^	2.5 ± 0.2^acef^	2.2 ± 0.6^abdef^	2.6 ± 0.09^abcef^	4.4 ± 0.2^abcdf^	4.1 ± 0.2^abcde^
Plantform	G	nt	nt	4.6 ± 0.9 4.7 ± 0.1**	nt	nt	nt

*nt* not tested

*See ‘Materials and methods’; **Gi of microshoots after elicitor (YE) treatment

^a^*p* < 0.05 vs. 10-day growth period

^b^*p* < 0.05 vs. 20-day growth period

^c^*p* < 0.05 vs. 30-day growth period

^d^*p* < 0.05 vs. 40-day growth period

^e^*p* < 0.05 vs. 50-day growth period

^f^*p* < 0.05 vs. 60-day growth period

The experiments on the accumulation of SL in SchS agar cultures were carried out on six variants of MS medium with different concentrations of PGRs: BA and NAA (variants B–G), and a medium containing no PGRs as control conditions (variant A). The PGRs applied in the tested variants of MS medium as well as the duration of the culture cycle significantly influenced the amounts of all the 14 lignans analysed in the cultured biomass.

Detailed data on the amounts of SL obtained on the tested MS media variants after each duration of the growth period is included in supplementary materials as Tables S1–S6 (Online resource). Based on the analyses, considerable differences in the amounts of individual compounds and in their total content were found depending on the combination of the PGRs used in the culture medium. The amounts of the individual SL varied within very wide limits, e.g. for schisandrin from 25.6 to 176.3 mg/100 g DW, for schisantherin B from 7.2 to 50.1 mg/100 g DW and for the total content from 125.3 to 490.3 mg/100 g DW (Table 4). Higher SL content was obtained in extracts from the biomass grown on variants B–G than in extracts of the biomass grown on variant A (Tables S1–S6). The quantitatively dominant lignans in extracts from the agar cultures were as follows: schisandrin (max. 176.3 mg/100 g DW, variant E, culture duration—30 days), angeloylgomisin Q (max. 85.1 mg/100 g DW, variant E, culture duration—50 days), schisantherin B (max. 50.1 mg/100 g DW, variant E, culture duration—10 days), gomisin A (max 49.6 mg/100 g DW, variant G, culture duration—30 days) and deoxyschisandrin (max. 34.1 mg/100 g DW, variant G, culture duration—30 days) (Table 4).

**Table 4:** The comparison of schisandra lignans yielding in *S. chinensis* cv. Sadova agar microshoot cultures—changes in dependence on applied duration of growth period and media variants. Results correspond to Tables 2 and S1–S6

Lignans	Content (mg/100 g DW) min.–max.	Increase (fold)	Optimal growth period (days)	Optimal medium variant*	Gi for optimal conditions
Schisandrin	25.6–176.3	6.9	30	E	3.1
Gomisin A	8.7–49.6	5.7	30	G	4.8
Angeloyl-/tigloylgomisin H	13.5–67.0	5.0	10	E	2.3
Angeloyl-/tigloylgomisin Q	10.8–85.1	7.9	50	E	6.1
Gomisin G	3.7–20.5	5.5	50	G	7.1
Schisantherin A	1.2–13.7	11.7	10	E	2.3
Schisantherin B	7.2–50.1	6.9	10	E	2.3
Schisanthenol	0.2–3.2	14.0	50	G	7.1
Deoxyschisandrin	12.0–34.1	2.9	30	G	4.8
Schisandrin B	3.1–27.4	8.7	40	G	6.8
γ-Schisandrin	1.6–22.8	14.7	60	D	6.3
Benzoylgomisin P	4.4–39.8	9.0	50	G	7.1
Schisandrin C	1.9–18.4	9.5	60	G	8.7
Schisantherin D	2.9–58.7	20.0	10	E	2.3
Total content	125.3–490.3	4.6	30	E	4.8

*See ‘Materials and methods’

**Fig. 5:**
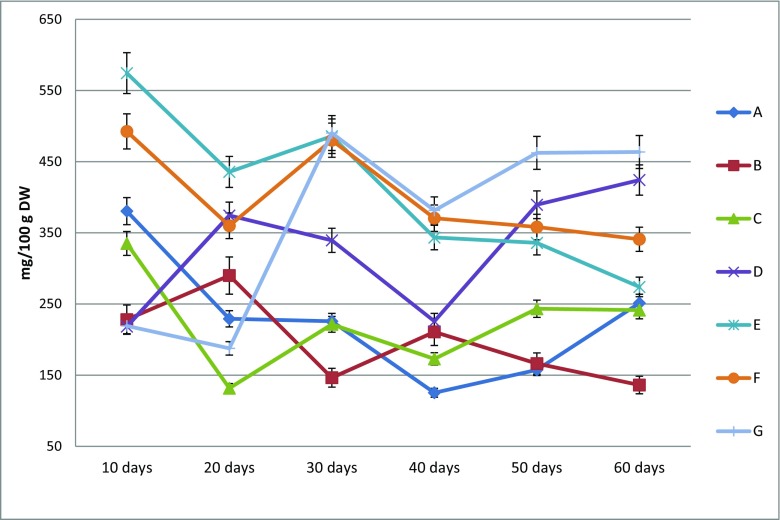
The dynamic of schisandra lignans accumulation in agar *S. chinensis* cv. Sadova microshoots cultures based on their total contents with relation to the duration of growth period and applied MS medium variants

The maximum total SL content (574.4 mg/100 g DW) was found after 10 days of growth on variant E of the culture medium. However, the biomass growth during this period was low (Gi = 2.3). High production of SL (490.3 mg/100 g DW) was also found on the same MS medium variant in extracts from the microshoots harvested after 30 days, for which the Gi factor was higher (Gi = 4.8) (Tables 4 and S3). After that period, the observed increase in SL in the microshoot cultures was the highest (Fig. 5). High total amounts of lignans were also obtained in the microshoots after 30 days of their cultivation on variants E and F of MS medium. The lowest total SL content was found after 20 days of culture on variant C. Greater amounts of lignans were obtained in biomasses grown on the test variants D, E, F and G than on the control medium, variant A (containing no PGRs). The results on variants B and C were comparable with the control (Table 4, Fig. 5).

#### Agitated microshoot cultures

The appearance of the biomass in the agitated shoot cultures depended on the variant of MS medium and the duration of the culture cycle. The largest increases in biomass were found after 60 days of culture on variant F, and the smallest after 10 days of culture on variant G (Fig. 2). Depending on the medium variant and culture duration, the obtained increases in dry biomass, expressed by the growth index (Gi), ranged from 1.4 to 7.5 times. The highest increases were obtained for cultures cultivated for 60 days on variant H (Table 3).

Based on the HPLC-DAD analyses, considerable differences were found in the amounts of the individual compounds as well as in their total amount, depending on the combination of PGRs used in the culture medium. The amounts of the individual SL varied within very wide limits, e.g. for schisandrin from 26.8 to 112.3 mg/100 g DW, for schisantherin B from 9.6 to 29.9 mg/100 g DW and for the total content from 112.0 to 414.8 mg/100 g DW (Table 5).

**Table 5:** The comparison of schisandra lignans yielding in *S. chinensis* cv. Sadova agitated microshoot cultures—changes in dependence on applied duration of growth period and media variants. Results correspond to Tables 2 and S7–S12

Lignans	Content (mg/100 g DW) min.–max.	Increase (fold)	Optimal growth period (days)	Optimal medium variant*	Gi for optimal conditions
Schisandrin	26.8–112.3	4.2	30	G	2.2
Gomisin A	17.6–71.4	4.1	30	G	2.2
Angeloyl-/tigloylgomisin H	11.9–44.8	3.8	30	G	2.2
Angeloyl-/tigloylgomisin Q	4.0–31.2	7.9	30	F	2.9
Gomisin G	1.3–9.5	7.2	20	G	2.5
Schisantherin A	1.4–7.5	5.2	30	G	2.2
Schisantherin B	9.6–29.9	3.1	30	G	2.2
Schisanthenol	0.7–8.4	11.6	30	G	2.2
Deoxyschisandrin	12.4–27.3	2.2	20	G	2.5
Schisandrin B	0.1–11.5	114.9	30	F	2.9
γ-Schisandrin	1.3–10.5	8.0	20	G	2.5
Benzoylgomisin P	2.4–22.0	9.2	30	F	2.9
Schisandrin C	0.5–6.1	11.4	20	F	2.3
Schisantherin D	2.4–22.3	9.2	30	G	2.2
Total content	120.0–414.8	3.5	30	G	2.2

*See ‘Materials and methods’

The quantitatively dominant SL in extracts from the biomass of the agitated cultures were as follows: schisandrin (max. 112.3 mg/100 g DW, variant G, culture duration—30 days), gomisin A (max. 71.4 mg/100 g DW, variant G, culture duration—30 days), angeloylgomisin H (max. 44.8 mg/100 g DW, variant G, culture duration—30 days), angeloylgomisin Q (max. 31.2 mg/100 g DW, variant F, culture duration—30 days), schisantherin B (max. 29.9 mg/100 g DW, variant G, culture duration—30 days) and deoxyschisandrin (max. 27.3 mg/100 g DW, variant G, culture duration—20 days) (Tables S7–S12, and Table 5). Extracts from the culture media were found to contain only trace amounts of SL (less than 0.1 mg/l).

**Fig. 6:**
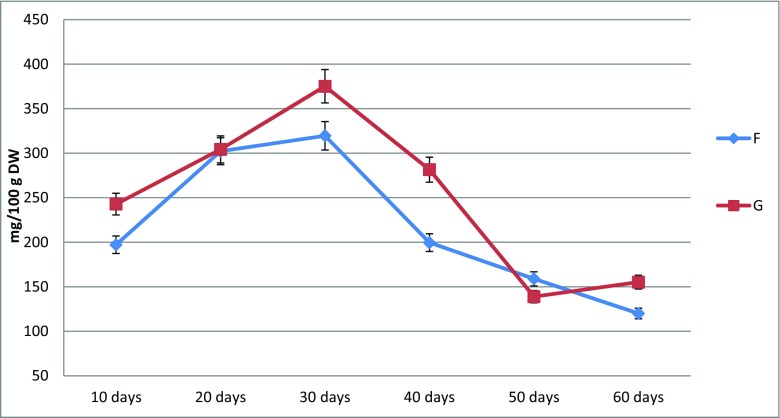
The dynamic of schisandra lignans accumulation in agitated *S. chinensis* cv. Sadova microshoots cultures based on their total contents with relation to the duration of growth period and applied MS medium variants

The maximum total SL content in extracts from agitated microshoot cultures was obtained after 30 days of cultivation on variant G (Table 5 and Fig. 6). The lowest total SL content was found after 60 days of cultivation on variant F medium. The 30-day growth period and application of variant G of MS medium yielded the most satisfactory results in terms of culture growth and SL accumulation (Fig. 6).

#### Bioreactor cultures

The best conditions determined on the basis of the results of SL accumulation obtained during the testing of agitated cultures were chosen for the bioreactor-grown microshoot cultures of SchS. The most favorable medium was the MS variant G and a 30-day growth period. Based on the observations of microshoot morphology, vital growth of tissue under cultivation in the temporary immersion system was found (Fig. 3). The growth increments, expressed as Gi, were high, equal to 4.6 (Table 6).

**Table 6:** Schisandra lignans contents (mg/100 g DW±SD) in extracts from microshoots *S. chinensis* cv. Sadova cultivated in Plantform temporary immersion system on MS variant G for 30-day growth periods (*n* = 3). I—microshoots without elicitation; II—microshoots elicitated with 1 g/l of YE (yeast extract) on the 20^th^ day of growth period

Lignans	I	II	Increase (fold)
Schisandrin	115.3 ± 1.4^b^	144.4 ± 2.8^a^	1.3
Gomisin A	27.9 ± 1.3^b^	42.6 ± 0.7^a^	1.5
Angeloyl-/tigloylgomisin H	30.0 ± 0.7^b^	45.1 ± 9.6^a^	1.5
Angeloyl-/tigloylgomisin Q	18.3 ± 0.2^b^	22.3 ± 0.7^a^	1.2
Gomisin G	9.5 ± 0.2^b^	12.0 ± 1.6^a^	1.3
Schisantherin A	4.1 ± 0.1^b^	5.0 ± 0.2^a^	1.2
Schisantherin B	35.6 ± 0.5^b^	44.2 ± 4.6^a^	1.2
Schisanthenol	1.1 ± 0.2	1.4 ± 0.5	1.4
Deoxyschisandrin	9.6 ± 0.2^b^	13.2 ± 1.1^a^	1.4
Schisandrin B	9.1 ± 0.2^b^	11.0 ± 0.4^a^	1.2
γ-Schisandrin	2.3 ± 0.1^b^	2.8 ± 0.1^a^	1.3
Benzoylgomisin P	12.3 ± 0.2^b^	15.4 ± 0.5^a^	1.3
Schisandrin C	16.6 ± 0.4^b^	20.8 ± 0.6^a^	1.3
Schisantherin D	22.0 ± 0.3^b^	28.2 ± 0.5^a^	1.3
Total content	313.5 ± 6.1^b^	408.3 ± 23.9^a^	1.3

^a^*p* < 0.05 vs. I—microshoots without elicitation

^b^*p* < 0.05 vs. II—microshoots elicitated with 1 g/l of YE (yeast extract) on the 20^th^ day of growth period

The dominant compounds in extracts from the microshoots not subjected to elicitation were as follows: schisandrin (115.3 mg/100 g DW), schisantherin B (35.6 mg/100 g DW) and angeloylgomisin H (30.1 mg/100 g DW) (Table 6). The total SL content was 313.5 mg/100 g DW.

The experiment with Plantform bioreactor-grown microshoots was expanded to include elicitor treatments using 1 g/l YE on day 20 of biomass growth. In extracts from the elicited microshoots, the amounts of the individual compounds increased from 1.22- to 1.53-fold (Table 6). The dominant compounds were as follows: schisandrin (144.3 mg/100 g DW), schisandrin B (44.2 mg/100 g DW), gomisin A (42.6 mg/100 g DW) and angeloylgomisin H (45.1 mg/100 g DW). The total content was 408.3 mg/100 g DW, which was 1.30 times higher than in extracts from the microshoots not subjected to elicitation (Table 6). The Plantform culture media were found to contain only trace amounts of SL (less than 0.1 mg/l).

## Discussion

The results of chromatographic analyses of SL in extracts from fruits and leaves of SchS showed that the qualitative composition of lignan fraction was the same as in the Sch plant matrices analysed by Szopa et al. (Szopa et al. 2016b). However, the two plants differed with respect to the amounts of individual compounds. Importantly, higher concentrations of the analysed SL were found in the schisandra cultivar. The amounts of SL in the extracts from fruits and leaves of SchS were 1.17- and 1.34-fold higher, respectively, than in the extracts from the basic species (Sch) which is widely distributed and utilized as a medicinal plant (Table 2).

The same lignans were dominant in extracts from the fruits of both plants, namely schisandrin, gomisin A, angeloylgomisin H and *γ*-schisandrin. However, the schisandrin and *γ*-schisandrin contents in SchS fruit extracts were respectively 1.26- and 1.45-fold higher, and those of gomisin A and angeloylgomisin H respectively 1.51- and 2.26-fold lower than in Sch fruit extracts. The schisantherin B content in SchS fruit extracts was 12-fold higher.

In extracts from the leaves of Sch and SchS, different lignans were shown to prevail. SchS leaf extracts were found to contain greater amounts of some of SL: 1.86 times more schisandrin and 4.02 times more schisantherin D (Table 2).

In the light of the presented comparative phytochemical studies, SchS proved to contain higher amounts of SL than the parent species (Sch). Given the fact that these compounds largely determine the therapeutic properties of *Schisandra* species, SchS can be proposed as a new raw material for pharmaceutical use. However, broader phytochemical studies are required to evaluate SchS for the presence of other biologically active metabolites like phenolics and polysaccharides. The results encourage further phytochemical studies on SchS.

Literature data confirm the fact that cultivated varieties of medicinal plant species are often more useful as a source of raw materials for pharmaceutical, cosmetic and dietary use. Cultivars are often characterized as being easier to grow, more resistant to external factors and more productive like *Aronia melanocarpa* cv. Galicjanka (Wangensteen et al. 2014), *Cannabis sativa* cv. Uso 11 (Sankari 2000), *Vitis vinifera* cvs. Palatina and Swenson Red (Elmer) (Corrales et al. 2008), or *Zea mays* cvs. Rugosa and Pioneer (Drew and Läuchli 1985). Many vegetable cultivars are characterized by high production of active ingredients—such as *Anethum graveolens* (26 various cvs.) with a high essential oil content (Bailer et al. 2001), *Ipomoea batatas* cvs. Lunyangwa, Semusa, Kenya, Zondeni and Mugamba with a high starch content (Tsakama et al. 2010), *Daucus carota* cvs. Kazan and Kamaran with a high carotenoid content (Czepa and Hofmann 2004). Levels of biologically active compounds have been studied in cultivars of other medicinal plants; analyses have demonstrated, for example, a high phenolic content and high radical scavenging potential of *Arnica montana* cv. Arbo (Spitaler et al. 2008) and *Matricaria chamomilla* cv. Bona (Ganzera et al. 2008), or high levels of isoflavones in *Glycine max* cv. Saeolkong (Lee et al. 2009). Our results for the cultivar of Chinese magnolia vine correspond with those findings.

Besides examining SchS raw materials for SL content, the current study involved establishing in vitro shoot cultures of the Sadova cultivar, with the aim of providing an alternative, sustainable source of the aforementioned compounds. So far, there have been only few reports on in vitro cultures of Sch, including the projects of the authors of this paper (Březinová et al. 2010; Kohda et al. 2012; Szopa and Ekiert 2012; Szopa et al. 2016b), whereas in vitro cultures of SchS has not been studied at all.

In general, microshoot cultures of SchS were shown to accumulate higher amounts of SL as compared with previous reports. For instance, Brezinová et al. (Březinová et al. 2010) estimated the following amounts of SL in embryogenic Sch cultures cultivated on a Westvaco agar medium (with 0.9 mg/l BA and 2.21 mg/l 2,4-D (2,4-dichlorophenoxyacetic acid)): *γ*-schisandrin (max. 13.9 mg/100 g DW), gomisin A (max. 11.9 mg/100 g DW), deoxyschisandrin (max. 9.6 mg/100 g DW), schisandrin C (max. 7.3 mg/100 g DW) and schisandrin (max. 0.9 mg/100 g DW). In comparison with these results, the amounts of SL obtained in our SchS agar microshoot cultures were respectively 1.64-, 4.16-, 3.54-, 2.51- and 195.90-fold higher (Table 3).

In a study conducted by Kohda et al. (Kohda et al. 2012), Sch callus cultures were grown on ½ MS medium enriched with kinetin (0.05 mg/l) and 2,4-D (0.2 mg/l). Only two of the six analysed lignans, namely gomisin A (max 0.05 mg/100 g DW) and gomisin F (max 0.04 mg/100 g DW), were found in the biomass. The amount of gomisin A obtained in extracts from our SchS agar microshoot cultures was 99.10 times higher (Table 4).

Comparing the results of this study on SchS with our previous work on Sch agar cultures (Szopa et al. 2016b), it was found that the qualitative composition of SL was the same, but the amounts of specific constituents were different. In SchS agar cultures, the amounts of the main SL, schisandrin, deoxyschisandrin and gomisin A, were respectively 3.15-, 1.12-, 1.91-fold greater than the amounts of these compounds obtained in Sch microshoots on the corresponding variants of the culture medium. Also, the present study found SchS microshoots to contain 62.61-, 32.71- and 14.26-fold greater amounts of schisantherin B, schisantherin A and gomisin G, respectively, in comparison with Sch. Moreover, in SchS cultures, the amounts of angeloylgomisin Q and angeloylgomisin H were respectively 1.66- and 2.51-fold greater than the amounts in Sch biomass. The maximum total SL content obtained in extracts from the biomass from SchS agar cultures was 2.41 times higher than in extracts from Sch agar cultures (Table 7).

**Table 7:** Comparison of schisandra lignans contents (mg/100 g DW±SD) in agar, agitated and Plantform bioreactor microshoots cultures of *S. chinensis* cv. Sadova and *S. chinensis* on MS medium variant G, for 30 days

Lignans	*S. chinensis* cv. Sadova			S. chinensis*		
	Agar	Agitated	Bioreactor	Agar	Agitated	Bioreactor
Schisandrin	99.0 ± 7.1^bcdef^	112.3 ± 6.8^ade^	115.3 ± 1.4^ade^	53.0 ± 0.2^abcef^	38.0 ± 2.4^abcdf^	118.6 ± 1.5^abde^
Gomisin A	49.6 ± 1.6^bcdef^	71.4 ± 6.9^acde^	27.9 ± 1.3^abdf^	25.9 ± 2.0^abf^	24.4 ± 0.2^abcf^	67.9 ± 2.6^abcde^
Angeloyl-/tigloylgomisin H	54.3 ± 12.3^bcde^	44.8 ± 1.9^acdef^	30.0 ± 0.7^abef^	26.7 ± 1.2^abcf^	22.0 ± 0.3^abcf^	53.5 ± 2.4^bcde^
Angeloyl-/tigloylgomisin Q	67.7 ± 2.6^bcdef^	30.6 ± 0.8^acdef^	18.3 ± 0.2^abdef^	51.2 ± 2.6^abcef^	34.6 ± 0.4^acdf^	100.1 ± 3.1^abcde^
Gomisin G	10.7 ± 0.2^bcdef^	6.5 ± 4.3^acdef^	9.5 ± 0.2^bdef^	1.4 ± 0.8^abcf^	1.4 ± 0.1^abcf^	2.7 ± 0.5^abcde^
Schisantherin A	8.0 ± 0.3^bcdef^	7.5 ± 0.2^cdef^	4.1 ± 0.1^abdef^	0.4 ± 0.2^abcef^	3.1 ± 0.1^abcde^	3.0 ± 0.1^abcd^
Schisantherin B	33.8 ± 1.0^bdef^	29.9 ± 1.4^acdef^	35.6 ± 0.5^bdef^	0.8 ± 0.1^abcef^	3.4 ± 0.1^abcdf^	9.9 ± 2.1^abcde^
Schisanthenol	2.2 ± 0.1^bcdef^	8.4 ± 1.9^acde^g	1.1 ± 0.2^abd^	0.5 ± 0.1^abcf^	1.0 ± 0.1^abd^	1.3 ± 0.3^abd^
Deoxyschisandrin	34.0 ± 0.8^bcdef^	21.5 ± 0.5^acdef^	9.6 ± 0.2^abdef^	30.3 ± 1.9^bcf^	27.6 ± 0.3^abcf^	77.7 ± 4.9^abcde^
Schisandrin B	22.4 ± 0.5^bcdef^	0.4 ± 0.2^acdef^	9.1 ± 0.2^abdef^	14.5 ± 1.4^abcef^	13.6 ± 0.2^abcdf^	39.7 ± 3.4^abcde^
γ-Schisandrin	16.8 ± 0.9^bcdef^	5.7 ± 0.8^acdef^	2.3 ± 0.1^abdef^	8.1 ± 0.3^abcef^	7.6 ± 0.2^abcf^	22.4 ± 0.2^abcdef^
Benzoylgomisin P	27.0 ± 2.5^bcdef^	9.0 ± 0.3^acdef^	12.3 ± 0.2^abdef^	17.5 ± 0.9^abcef^	13.7 ± 0.1^abdf^	37.2 ± 0.9^abcde^
Schisandrin C	12.4 ± 0.5^bcdef^	4.9 ± 0.2^acdef^	16.6 ± 0.4^abdef^	2.4 ± 0.5^abcf^	2.0 ± 0.1^abcf^	5.1 ± 0.8^acde^
Schisantherin D	52.4 ± 1.2^bcdef^	22.3 ± 0.5^adef^	22.0 ± 0.3^adef^	5.3 ± 0.9^abcef^	3.0 ± 0.2^abcdf^	8.2 ± 0.8^abcde^
Total content	490.3 ± 31.5^bcdef^	375.1 ± 26.6^acdef^	313.5 ± 6.1^abdef^	237.9 ± 4.5^abcef^	195.2 ± 9.5^abcdf^	547.0 ± 12.5^abcde^

*According to Szopa et al. 2017b

^a^*p* < 0.05 vs. *S. chinensis* cv. Sadova agar in vitro cultures

^b^*p* < 0.05 vs. *S. chinensis* cv. Sadova agitated in vitro cultures

^c^*p* < 0.05 vs. *S. chinensis* cv. Sadova bioreactor in vitro cultures

^d^*p* < 0.05 vs. *S. chinensis* agar in vitro cultures

^e^*p* < 0.05 vs. *S. chinensis* agitated in vitro cultures

^f^*p* < 0.05 vs. *S. chinensis* bioreactor in vitro cultures

In the study by Brezinová et al. (Březinová et al. 2010) conducted on Sch suspension cultures, the dominant compounds were gomisin N (max. 54.7 mg/100 g DW), *γ*-schisandrin (max. 55.0 mg/100 g DW) and schisandrin C (max. 20.9 mg/100 g DW) whereas deoxyschisandrin (4.9 mg/100 g DW), gomisin A (max. 4.4 mg/100 g DW) and schisandrin (max. 1.0 mg/100 g DW) accumulated in smaller amounts. The maximum concentrations of *γ*-schisandrin and schisandrin C in extracts from the biomass of SchS agitated cultures were respectively 5.23 and 3.41 times smaller, and those of deoxyschisandrin 5.57 times greater than in extracts from the biomass of Sch suspension cultures. The current study also found 112.31- and 16.23-fold higher levels of schisandrin and gomisin A, respectively (Table 7).

The comparison of our studies on agitated microshoot cultures of SchS and Sch demonstrated that both plants have the same qualitative composition of SL; however, differences were shown in the amounts of the specific lignans. The dominant compounds in Sch agitated cultures were schisandrin, deoxyschisandrin and gomisin A. The maximum amounts of schisandrin and gomisin A were respectively 2.66 and 2.58 times lower, and those of deoxyschisandrin 1.31 times higher than the amounts of these compounds obtained in SchS agitated cultures. The amounts of schisanthenol, schisantherin B and gomisin G obtained in extracts from SchS agitated cultures were respectively 6.53-, 5.24- and 4.98-fold higher compared with agitated cultures of *S*. *chinensis*. The amounts of angeloylgomisin Q and angeloylgomisin H in Sch agitated microshoot cultures were 1.42 times higher and 1.70 times lower, respectively, in comparison with SchS. Also, the amounts of schisantherin D and schisandrin B obtained with SchS were 4.27 and 2.02 times greater, respectively, than in Sch agitated cultures. The maximum total SL content obtained in extracts from the biomass from SchS agitated cultures was 1.69 times higher than in extracts from Sch agitated cultures (Table 7).

The bioreactor-grown SchS microshoots, in comparison with Sch microshoots cultivated under the same conditions (Szopa et al. 2017b), were found to contain greater amounts of three main SL: schisantherin B (3.62 times), schisandrin C (3.32 times) and schisantherin D (2.70 times) (Table 7). However, the total SL content in Plantform-grown SchS was 1.74 times lower than in Sch.

The elicitation experiment performed on Plantform-grown microshoots of SchS increased the total amount of SL 1.30 times; however, the obtained amounts were still lower than in non-elicited Sch microshoots grown in the same temporary-immersion system (Szopa et al. 2018). The applied elicitation scheme, taken directly from our previous report, may not be optimal for in vitro shoots of *Schisandra* cultivar. Further optimization of the experiment is required to determine the type, concentration and application time of the elicitor, as well as the cultivation mode providing highest SL yield. Indeed, our previous trials on Sch in vitro cultures had indicated that the amounts of SL in the microshoots vary considerably depending on bioreactor type (Szopa et al. 2017b) and elicitation scheme (Szopa et al. 2018). Moreover, as it has been demonstrated with other in vitro cultures of medicinal plant species, like *Panax ginseng* (Thanh et al. 2005), *Taxus chinensis* (Dong and Zhong 2002) and *Withania somnifera* (Sivanandhan et al. 2014), these conditions must be designed for each types of in vitro cultures and plant species individually.

As a result of the experiments with SchS, a cultivar of Sch, quantitative differences in the chemical composition in vitro cultures of different cultivars were proven. Similar correlations of the diversity in the characteristics of cultivated varieties have been confirmed by studies conducted on other cultures in vitro. These differences are most often presented in reports on micropropagation. The necessity of introducing different schemes for dealing with cultivars of the same species have been demonstrated for *Hibiscus cannabinus* cv. Guangdong 743-2 (Ayadi et al. 2011), *Pyrus communis* cv. Cascatense (De Castro Da Silva et al. 2016) and *Vitis vinifera* cv. Cabernet Sauvignon (Laslo et al. 2010). Particularly noteworthy are studies on the accumulation of biologically active metabolites in the biomass of in vitro cultures of cultivated varieties such as in vitro cultures of three cultivars of *Hypericum perforatum*—Elixir, Helos and Topas (Kwiecień et al. 2015; Kwiecień et al. 2018). The richest source of the estimated compounds, i.e. phenolic acids and flavonoids, and hence a potential biotechnological platform, were the shoots of cv. Helos cultivated in an agitated culture system on LS and MS media containing low concentrations of BA and NAA (0.1–1.0 mg/l). Variability in the accumulation of free proline has been observed in different cultivars of *Phaseolus vulgaris* cvs. Pinto Americano, Pastilla, Flor de Mayo and Flor de Junio (Cárdenas-Avila et al. 2006). The most promising results were confirmed for cv. Pinto Americano.

In recent decades, in vitro cultures of several medicinal plants were investigated as an alternative for traditional crops or wild-grown species. This type of studies are boosted by limited natural resources of medicinal plants which are not sufficient to meet the growing market demands. Examples of successful research in this field include in vitro cultures of *Catharanthus roseus* (Vázquez-Flota et al. 2009), *Panax ginseng* (Thanh et al. 2005) or *Salvia miltiorrhiza* (Zhao et al. 2010), which were employed for the production of biomass and/or bioactive compounds of interest. In the presented work, in vitro cultures of SchS were evaluated for their utility as a sustainable source of SL. The maximal total amounts of SL obtained through testing different culture conditions (490.3 mg/100 g DW) was 2.04 times higher than in leaf extract and only 1.32 times lower than in the fruit extract od parent plant (Tables 2 and 7). Also the individual contents of some SL were higher in the microshoot extracts than in analysed for comparative purpose fruit and leaf extracts of soil-grown plant. The amounts of the main SL with high therapeutic value were respectively 1.45 and 2.09 (schisandrin) and 2.23 and 5.63 (gomisin B) times higher in extracts from in vitro cultures. Since the above compounds are considered to be responsible for biological activity of *Schisandra* extracts (Szopa et al. 2017a), the obtained cultures may serve as a material for their isolation. In the course of the study, we have also optimized the growth conditions of SchS microshoots in the temporary immersion system (Plantform bioreactors) which enable effective propagation of microshoots with relatively low cost (Szopa et al. 2017b). These experiments included the stimulation of SL biosynthesis by elicitation, thus providing an opportunity for the scale up the production of SL-enriched biomass (Table 6). Promising results encourage further experiments in the field. Most importantly, our findings demonstrated that SchS is a rich source of SL, thus proving its value for medical, cosmetic and food industry.

The chromatographic analyses of all analysed SL standards (nine compounds) performed under our study was confirmed by validation procedure compatible with European Medicines Agency standards based on accuracy, precision, linearity, limit of detection and limit of quantification (European Medicines Agency 2005) (see section ‘Validation setup’).

Within the framework of our phytochemical and what is the most important, biotechnological studies of a cultivar of Sch, our results indicated, for the first time, the highest utility of SchS with regard to higher concentrations of SL. These compounds are responsible for a wide range of biological properties, especially of fruits, which determine their application in medicine, phytotherapy and cosmetology industry (Walsh 2003; Schneider et al. 2013).

## Electronic supplementary material

ESM 1 (PDF 693 kb)
