# Supplementary material for: Phytochemical and biotechnological studies on Schisandra chinensis cultivar Sadova No. 1—a high utility medicinal plant
Source: Appl Microbiol Biotechnol. 2018 Apr 23;102(12):5105–20. doi: 10.1007/s00253-018-8981-x (PMC5959991; doi:10.1007/s00253-018-8981-x)
Supplement: Supplementary file 1 — (PDF 693 kb) [file 253_2018_8981_MOESM1_ESM.pdf]

**<u>Applied Microbiology and Biotechnology</u>**

original article

# Phytochemical and biotechnological studies on *Schisandra chinensis* cultivar Sadova No. 1 – a high utility medicinal plant

Szopa Agnieszka*[1], Klimek-Szczykutowicz Marta[1], Kokotkiewicz Adam[2], Maślanka Anna[3], Agata Król[2], Luczkiewicz Maria[2], Ekiert Halina[1]

[1] *Chair and Department of Pharmaceutical Botany, Jagiellonian University, Medical College, Faculty of Pharmacy, ul. Medyczna 9, 30-688 Kraków, Poland, e-mail:a.szopa@uj.edu.pl*

[2] *Chair and Department of Pharmacognosy, Faculty of Pharmacy, Medical University of Gdansk, al. gen. J. Hallera 107, 80-416 Gdańsk, Poland*

[3] *Department of Inorganic and Analytical Chemistry, Jagiellonian University, Medical College, Faculty of Pharmacy, ul. Medyczna 9, 30-688 Kraków, Poland*

*Corresponding author:
phone +48 12 620 54 30, fax +48 620 54 40, e-mail: a.szopa@uj.edu.pl

Schisandrin

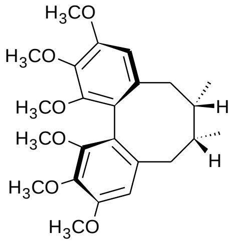

Gomisin A

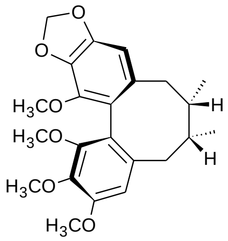

Deoxyschisandrin

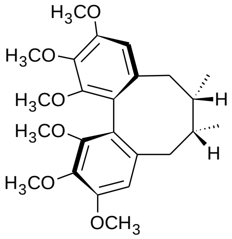

Schisandrin B

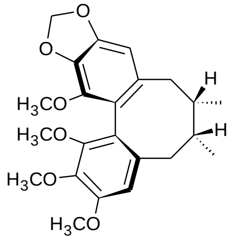

Schisandrin C

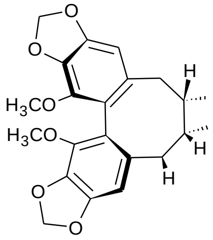

γ-Schisandrin

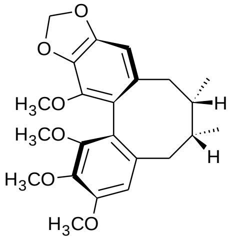

Schisanthenol

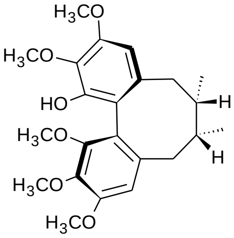

Schisantherin A

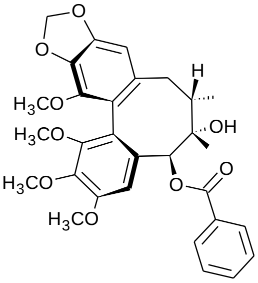

Schisantherin B

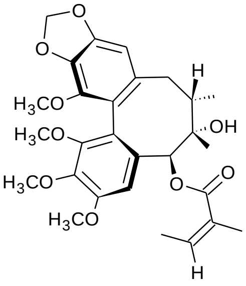

Figure S1. The chemical structures of main schisandra lignans (acc Szopa et al. 2017a).

Table S1. Accumulation [mg/100 g DW ± SD] of schisandra lignans in agar microshoot *Schisandra chinensis* cv. Sadova cultures cultured on different MS medium variants after 10 days growth period. Different letters indicate significant differences between means (n = 3, $p<0.05$).

| **Lignans** | **MS medium variants*** | | | | | | |
|---|---|---|---|---|---|---|---|
| | **A** | **B** | **C** | **D** | **E** | **F** | **G** |
| **Schisandrin** | 144.0±1.7 | 51.1±1.5 | 79.9±2.6 | 76.7±1.7 | 173.7±5.1 | 141.9±7.7 | 76.4±1.0 |
| **Gomisin A** | 23.9± 0.5 | 23.0±0.5 | 32.6±0.8 | 16.3±0.4 | 42.1±2.3 | 43.8±1.7 | 13.4±2.3 |
| **Angeloyl-/tigloylgomisin H** | 47.9±1.2 | 28.7±2.0 | 43.5±1.1 | 26.9±1.2 | 67.0±2.2 | 57.9±3.6 | 26.0±4.2 |
| **Angeloyl-/tigloylgomisin Q** | 26.1±0.6 | 51.9±1.2 | 56.9±1.7 | 24.8±2.3 | 63.9±3.7 | 58.9±4.4 | 22.2±1.4 |
| **Gomisin G** | 8.6±0.1 | 8.5±0.1 | 11.6±0.5 | 6.3±0.4 | 12.9±1.1 | 14.5±0.9 | 6.3±0.2 |
| **Schisantherin A** | 7.8 ±0.8 | 1.6±0.1 | 3.6±0.1 | 1.9±0.1 | 13.7±1.1 | 9.7±0.9 | 3.0±0.1 |
| **Schisantherin B** | 32.1±0.4 | 9.2±0.1 | 17.9±0.9 | 14.3±2.1 | 50.1±2.9 | 36.0±0.8 | 18.2±0.7 |
| **Schisanthenol** | 0.9±0.1 | 0.6±0.1 | 2.3±0.1 | 0.8±0.1 | 1.2±0.2 | 1.4±0.1 | 0.8±0.1 |
| **Deoxyschisandrin** | 23.6±0.9 | 13.7±0.1 | 15.8±0.5 | 14.4±0.2 | 30.2±1.3 | 25.5±1.2 | 15.6±0.3 |
| **Schisandrin B** | 10.0±0.5 | 6.3±0.1 | 10.4±0.6 | 5.3±0.1 | 12.4±1.2 | 11.6±1.5 | 5.6±0.3 |
| **γ-Schisandrin** | 8.2±0.5 | 8.0±0.2 | 12.3±0.8 | 6.9±0.4 | 12.7±0.9 | 12.4±1.8 | 6.5±0.3 |
| **Benzoylgomisin P** | 11.5±0.2 | 16.4±0.3 | 21.7±0.9 | 11.6±1.5 | 23.7±0.7 | 24.6±1.1 | 11.5±0.7 |
| **Schisandrin C** | 7.4±0.8 | 5.2±0.1 | 12.3±0.7 | 4.3±1.3 | 12.0±1.2 | 12.1±1.3 | 4.4±0.4 |
| **Schisantherin D** | 28.6±2.0 | 4.2±0.1 | 14.3±0.3 | 7.9±0.4 | 58.7±2.5 | 42.3±1.8 | 9.5±0.9 |
| **Total content** | **380.5±10.3**$^{bcdefg}$ | **228.1±6.4**$^{acdef}$ | **335.1±11.5**$^{abdefg}$ | **218.4±12.0**$^{abcef}$ | **574.4±26.4**$^{abcdfg}$ | **492.6±28.7**$^{abcdeg}$ | **219.3±12.9**$^{abcef}$ |

*A- control (without PGRs), B- 0.1 mg/l BA and 2 mg/l NAA, C- 0.5 mg/l BA and 2 mg/l NAA, D- 2 mg/l BA and 0,5 mg/l NAA, E- 2 mg/l BA and 1 mg/l NAA, F- 2 mg/l BA and 2 mg/l NAA, G- 3 mg/l BA and 1 mg/l NAA

$^{a}$ $p<0.05$ *vs*. MS medium variant A; $^{b}$ $p<0.05$ *vs*. MS medium variant B; $^{c}$ $p<0.05$ *vs*. MS medium variant C; $^{d}$ $p<0.05$ *vs*. MS medium variant D; $^{e}$ $p<0.05$ *vs*. MS medium variant E; $^{f}$ $p<0.05$ *vs*. MS medium variant F; $^{g}$ $p<0.05$ *vs*. MS medium variant G

Table S2. Accumulation [mg/100 g DW ± SD] of schisandra lignans in agar microshoot *S. chinensis* cv. Sadova cultures cultured on different MS medium variants after 20 days growth period. Different letters indicate significant differences between means (n = 3, $p<0.05$).

| **Lignans** | **MS medium variants*** | | | | | | |
|---|---|---|---|---|---|---|---|
| | **A** | **B** | **C** | **D** | **E** | **F** | **G** |
| **Schisandrin** | 83.0±10.0 | 75.6±2.0 | 25.6±18.0 | 119.2±7.1 | 111.5±5.7 | 121.2±8.6 | 55.2±1.4 |
| **Gomisin A** | 18.4±1.3 | 26.1±0.6 | 17.5±0.8 | 32.3±0.6 | 40.4±0.9 | 27.6±0.3 | 17.1±1.0 |
| **Angeloyl-/tigloylgomisin H** | 19.9±0.8 | 32.7±5.2 | 13.5±0.4 | 44.5±1.6 | 47.5±3.0 | 43.3±0.2 | 21.9±2.1 |
| **Angeloyl-/tigloylgomisin Q** | 22.3±1.4 | 53.7±0.6 | 16.9±0.6 | 44.2±1.4 | 74.9±1.3 | 24.9±1.5 | 23.2±1.7 |
| **Gomisin G** | 8.3±0.7 | 9.0±0.1 | 3.9±0.1 | 11.6±0.2 | 12.7±0.1 | 7.5±0.5 | 7.1±0.3 |
| **Schisantherin A** | 4.0±0.1 | 2.1±0.1 | 1.4±0.1 | 2.4±0.2 | 8.8±0.7 | 8.4±0.2 | 2.1±0.1 |
| **Schisantherin B** | 14.3±2.3 | 12.5±0.2 | 7.6±0.3 | 24.5±0.5 | 25.4±1.2 | 30.3±1.0 | 13.2±1.2 |
| **Schisanthenol** | 0.7±0.1 | 0.9±0.1 | 0.8±0.1 | 1.6±0.1 | 1.1±0.1 | 1.0±0.1 | 0.8±0.1 |
| **Deoxyschisandrin** | 16.1±0.3 | 18.0±0.1 | 17.9±0.3 | 20.2±0.2 | 22.7±0.4 | 22.3±1.0 | 13.8±0.2 |
| **Schisandrin B** | 8.7±0.2 | 9.0±0.1 | 8.7±1.5 | 18.4±1.0 | 11.2±0.6 | 6.9±0.1 | 4.5±0.1 |
| **γ-Schisandrin** | 5.3±0.3 | 12.0±0.3 | 6.1±0.2 | 17.0±0.7 | 11.6±0.7 | 8.5±0.3 | 6.3±0.3 |
| **Benzoylgomisin P** | 9.5±0.9 | 24.4±1.7 | 5.7±0.4 | 20.0±1.5 | 24.4±3.0 | 14.4±0.6 | 11.5±0.7 |
| **Schisandrin C** | 5.1±0.2 | 6.7±0.3 | 2.0±0.2 | 9.0±0.7 | 10.9±0.3 | 9.6±0.7 | 4.6±0.3 |
| **Schisantherin D** | 13.7±0.5 | 7.4±0.1 | 4.0±0.1 | 9.6±0.2 | 32.8±1.1 | 34.2±2.0 | 6.5±0.2 |
| **Total content** | **229.2±18.9**[bcdefg] | **290.0±11.4**[acdefg] | **131.7±23.0**[abdefg] | **374.5±15.9**[abcefg] | **435.7±19.1**[abcdfg] | **359.95±16.45**[abcdeg] | **187.7±9.4**[abcdef] |

*A- control (without PGRs), B- 0.1 mg/l BA and 2 mg/l NAA, C- 0.5 mg/l BA and 2 mg/l NAA, D- 2 mg/l BA and 0.5 mg/l NAA, E- 2 mg/l BA and 1 mg/l NAA, F- 2 mg/l BA and 2 mg/l NAA, G- 3 mg/l BA and 1 mg/l NAA

[a] $p<0.05$ *vs*. MS medium variant A; [b] $p<0.05$ *vs*. MS medium variant B; [c] $p<0.05$ *vs*. MS medium variant C; [d] $p<0.05$ *vs*. MS medium variant D; [e] $p<0.05$ *vs*. MS medium variant E; [f] $p<0.05$ *vs*. MS medium variant F; [g] $p<0.05$ *vs*. MS medium variant G

Table S3. Accumulation [mg/100 g DW ± SD] of schisandra lignans in agar microshoot *S. chinensis* cv. Sadova cultures cultured on different MS medium variants after 30 days growth period. Different letters indicate significant differences between means (n = 3, $p<0.05$).

| **Lignans** | **MS medium variants*** | | | | | | |
|---|---|---|---|---|---|---|---|
| | **A** | **B** | **C** | **D** | **E** | **F** | **G** |
| **Schisandrin** | 81.4±2.3 | 39.1±0.7 | 37.5±0.8 | 115.1±4.7 | 176.3±6.0 | 163.3±4.0 | 99.0±7.1 |
| **Gomisin A** | 14.7±0.9 | 15.3±0.4 | 25.4±0.9 | 25.6±0.6 | 35.5±3.2 | 39.8±1.4 | 49.6±1.6 |
| **Angeloyl-/tigloylgomisin H** | 28.2±2.4 | 17.4±0.6 | 29.0±2.0 | 40.9±1.2 | 58.2±1.2 | 54.8±1.5 | 54.3±12.3 |
| **Angeloyl-/tigloylgomisin Q** | 29.0±0.6 | 15.6±0.3 | 51.8±2.3 | 40.6±1.4 | 31.1±1.9 | 35.8±2.4 | 67.7±2.6 |
| **Gomisin G** | 6.2±0.1 | 3.7±0.1 | 11.2±0.5 | 10.9±0.5 | 11.5±1.3 | 14.1±0.7 | 10.7±0.2 |
| **Schisantherin A** | 1.5±0.1 | 1.3±0.1 | 1.5±0.2 | 2.0±0.1 | 11.0±1.1 | 10.4±0.6 | 8.0±0.3 |
| **Schisantherin B** | 15.4±0.4 | 9.6±0.2 | 8.0±0.2 | 19.8±1.1 | 49.6±2.1 | 38.0±1.9 | 33.8±1.0 |
| **Schisanthenol** | 0.6±0.1 | 0.8±0.1 | 1.0±0.1 | 1.3±0.2 | 0.9±0.1 | 1.6±0.1 | 2.2±0.1 |
| **Deoxyschisandrin** | 14.2±0.2 | 14.2±0.1 | 12.8±0.1 | 18.3±0.7 | 23.6±1.3 | 24.4±0.7 | 34.0±0.8 |
| **Schisandrin B** | 5.1±0.2 | 8.8±0.3 | 6.0±0.2 | 15.6±0.9 | 7.4±0.3 | 14.6±0.3 | 22.4±0.5 |
| **γ-Schisandrin** | 7.4±0.3 | 5.2±0.4 | 7.4±0.3 | 15.0±0.7 | 8.3±0.5 | 12.5±1.4 | 16.8±0.9 |
| **Benzoylgomisin P** | 12.3±0.5 | 9.3±2.3 | 17.0±0.8 | 17.1±1.1 | 15.2±1.2 | 18.4±0.7 | 27.0±2.5 |
| **Schisandrin C** | 4.0±0.2 | 2.6±0.5 | 8.2±0.4 | 9.1±0.7 | 11.1±0.5 | 11.5±0.2 | 12.4±0.5 |
| **Schisantherin D** | 5.8±0.4 | 3.7±0.2 | 4.7±0.2 | 8.2±0.9 | 46.2±0.8 | 40.9±1.1 | 52.4±1.2 |
| **Total content** | **225.7±8.1**$^{bdefg}$ | **146.5±6.0**$^{acdefg}$ | **221.4±8.8**$^{bdefg}$ | **339.4±14.5**$^{abcefg}$ | **485.7±21.5**$^{abcd}$ | **480.2±17.1**$^{abcdf}$ | **490.3±31.5**$^{abcde}$ |

*A- control (without PGRs), B- 0.1 mg/l BA and 2 mg/l NAA, C- 0.5 mg/l BA and 2 mg/l NAA, D- 2 mg/l BA and 0,5 mg/l NAA, E- 2 mg/l BA and 1 mg/l NAA, F- 2 mg/l BA and 2 mg/l NAA, G- 3 mg/l BA and 1 mg/l NAA

[a] $p<0.05$ *vs*. MS medium variant A; [b] $p<0.05$ *vs*. MS medium variant B; [c] $p<0.05$ *vs*. MS medium variant C; [d] $p<0.05$ *vs*. MS medium variant D; [e] $p<0.05$ *vs*. MS medium variant E; [f] $p<0.05$ *vs*. MS medium variant F; [g] $p<0.05$ *vs*. MS medium variant G

Table S4. Accumulation [mg/100 g DW ± SD] of schisandra lignans in agar microshoot *S. chinensis* cv. Sadova cultures cultured on different MS medium variants after 40 days growth period. Different letters indicate significant differences between means (n = 3, $p<0.05$).

| **Lignans** | **MS medium variants*** | | | | | | |
|---|---|---|---|---|---|---|---|
| | **A** | **B** | **C** | **D** | **E** | **F** | **G** |
| **Schisandrin** | 40.1±1.3 | 38.8±2.4 | 58.3±0.7 | 96.6±5.0 | 85.8±1.2 | 102.1±2.4 | 86.6±0.4 |
| **Gomisin A** | 12.5±0.3 | 25.6±0.5 | 13.9±1.0 | 18.4±0.4 | 31.6±0.8 | 36.4±0.5 | 42.0±0.6 |
| **Angeloyl-/tigloylgomisin H** | 14.3±0.5 | 18.5±0.4 | 20.6±1.1 | 16.4±0.2 | 39.3±2.4 | 47.8±2.9 | 28.9±2.1 |
| **Angeloyl-/tigloylgomisin Q** | 8.8±0.1 | 48.6±2.3 | 10.8±0.8 | 23.2±1.9 | 42.0±3.9 | 47.0±3.6 | 73.3±1.5 |
| **Gomisin G** | 4.4±0.1 | 8.4±0.2 | 4.4±0.1 | 4.8±0.1 | 9.9±0.2 | 12.7±0.5 | 12.0±0.5 |
| **Schisantherin A** | 2.1±0.1 | 1.9±0.1 | 3.0±0.1 | 2.2±0.1 | 7.6±0.4 | 5.5±0.1 | 2.6±0.1 |
| **Schisantherin B** | 10.6±0.3 | 7.2±0.2 | 16.0±0.7 | 8.6±0.5 | 26.3±0.3 | 29.5±0.2 | 14.5±1.8 |
| **Schisanthenol** | 0.4±0.1 | 1.0±0.1 | 0.7±0.1 | 0.8±0.1 | 1.1±0.1 | 1.1±0.1 | 1.6±0.1 |
| **Deoxyschisandrin** | 13.0±0.1 | 16.2±0.2 | 15.6±0.4 | 17.9±0.2 | 21.3±0.4 | 18.1±0.2 | 31.6±1.1 |
| **Schisandrin B** | 5.0±0.1 | 8.6±0.2 | 4.8±0.5 | 10.3±0.5 | 9.6±2.0 | 9.3±0.2 | 27.4±2.7 |
| **γ-Schisandrin** | 1.6±0.1 | 8.6±0.3 | 3.1±0.1 | 6.2±0.2 | 8.5±0.6 | 11.1±0.6 | 18.2±1.0 |
| **Benzoylgomisin P** | 4.4±0.3 | 17.3±1.0 | 5.2±0.1 | 8.1±0.2 | 20.0±0.7 | 19.3±1.3 | 25.8±3.4 |
| **Schisandrin C** | 1.9±0.2 | 5.4±0.2 | 4.2±0.1 | 3.6±0.1 | 9.5±0.8 | 10.3±0.3 | 10.5±0.7 |
| **Schisantherin D** | 6.3±0.1 | 4.5±0.1 | 12.5±0.3 | 8.4±0.1 | 30.8±0.7 | 20.5±1.7 | 6.5±0.2 |
| **Total content** | **125.3±3.5**$^{bcdefg}$ | **210.8±7.9**$^{acdefg}$ | **172.9±5.9**$^{abdefg}$ | **225.7±9.4**$^{abcefg}$ | **343.4±14.4**$^{abcdfg}$ | **370.6±14.3**$^{abcdeg}$ | **381.5±16.0**$^{abcdef}$ |

*A- control (without PGRs), B- 0.1 mg/l BA and 2 mg/l NAA, C- 0,5 mg/l BA and 2 mg/l NAA, D- 2 mg/l BA and 0.5 mg/l NAA, E- 2 mg/l BA and 1 mg/l NAA, F- 2 mg/l BA and 2 mg/l NAA, G- 3 mg/l BA and 1 mg/l NAA

[a] $p<0.05$ *vs*. MS medium variant A; [b] $p<0.05$ *vs*. MS medium variant B; [c] $p<0.05$ *vs*. MS medium variant C; [d] $p<0.05$ *vs*. MS medium variant D; [e] $p<0.05$ *vs*. MS medium variant E; [f] $p<0.05$ *vs*. MS medium variant F; [g] $p<0.05$ *vs*. MS medium variant G

Table S5. Accumulation [mg/100 g DW ± SD] of schisandra lignans in agar microshoot *S. chinensis* cv. Sadova cultures cultured on different MS medium variants after 50 days growth period. Different letters indicate significant differences between means (n = 3, $p<0.05$).

| **Lignans** | **MS medium variants*** | | | | | | |
|---|---|---|---|---|---|---|---|
| | **A** | **B** | **C** | **D** | **E** | **F** | **G** |
| **Schisandrin** | 52.3±0.8 | 54.5±0.8 | 42.7±1.2 | 138.9±2.4 | 51.6±1.3 | 104.2±5.2 | 88.9±0.2 |
| **Gomisin A** | 12.5±0.5 | 11.6±0.8 | 33.0±1.1 | 27.0±0.6 | 41.2±1.5 | 34.0±0.3 | 49.6±1.6 |
| **Angeloyl-/tigloylgomisin H** | 18.8±0.4 | 19.8±0.6 | 28.9±1.8 | 48.7±1.1 | 41.7±1.4 | 43.8±1.1 | 57.7±0.9 |
| **Angeloyl-/tigloylgomisin Q** | 16.3±0.3 | 19.6±0.6 | 47.0±1.1 | 38.0±1.7 | 85.1±1.5 | 42.1±1.2 | 76.0±7.1 |
| **Gomisin G** | 4.2±0.2 | 5.5±0.1 | 11.4±0.3 | 10.4±0.2 | 14.6±1.0 | 12.7±0.1 | 20.5±0.4 |
| **Schisantherin A** | 1.7±0.1 | 1.3±0.1 | 2.0±0.1 | 3.8±0.2 | 2.7±0.1 | 6.0±0.2 | 9.2±0.1 |
| **Schisantherin B** | 10.8±0.1 | 11.2±0.5 | 11.9±0.3 | 22.1±1.1 | 11.6±0.1 | 27.7±0.8 | 33.4±1.1 |
| **Schisanthenol** | 0.7±0.1 | 0.6±0.1 | 1.2±0.1 | 1.0±0.1 | 1.1±0.2 | 1.2±0.1 | 3.2±0.1 |
| **Deoxyschisandrin** | 13.3±0.1 | 13.6±0.1 | 15.4±0.2 | 21.7±1.1 | 14.1±0.2 | 17.8±0.1 | 20.5±1.1 |
| **Schisandrin B** | 6.8±0.1 | 4.0±0.1 | 11.9±0.8 | 10.3±0.6 | 10.5±0.3 | 10.4±0.6 | 17.4±1.9 |
| **γ-Schisandrin** | 5.5±0.1 | 6.3±0.2 | 9.3±0.5 | 14.7±0.4 | 12.5±1.8 | 8.8±0.6 | 19.9±0.9 |
| **Benzoylgomisin P** | 7.8±0.2 | 11.0±0.5 | 15.1±0.5 | 17.7±0.6 | 29.1±1.7 | 17.2±1.2 | 39.8±2.0 |
| **Schisandrin C** | 2.6±0.1 | 3.2±0.1 | 7.7±0.3 | 11.6±0.8 | 11.9±0.4 | 10.1±1.1 | 15.2±2.0 |
| **Schisantherin D** | 4.2±0.3 | 4.3±0.2 | 5.7±0.4 | 23.7±2.4 | 8.2±0.2 | 22.3±1.3 | 11.1±1.0 |
| **Total content** | **157.4±3.1**$^{cdefg}$ | **166.3±4.5**$^{cdefg}$ | **243.3±8.5**$^{abdefg}$ | **389.5±13.1**$^{abcefg}$ | **335.9±11.5**$^{abcdfg}$ | **358.2±13.9**$^{abcdeg}$ | **462.4±20.3**$^{abcdef}$ |

*A- control (without PGRs), B- 0.1 mg/l BA and 2 mg/l NAA, C- 0.5 mg/l BA and 2 mg/l NAA, D- 2 mg/l BA and 0,5 mg/l NAA, E- 2 mg/l BA and 1 mg/l NAA, F- 2 mg/l BA and 2 mg/l NAA, G- 3 mg/l BA and 1 mg/l NAA

[a] $p<0.05$ *vs*. MS medium variant A; [b] $p<0.05$ *vs*. MS medium variant B; [c] $p<0.05$ *vs*. MS medium variant C; [d] $p<0.05$ *vs*. MS medium variant D; [e] $p<0.05$ *vs*. MS medium variant E; [f] $p<0.05$ *vs*. MS medium variant F; [g] $p<0.05$ *vs*. MS medium variant G

Table S6. Accumulation [mg/100 g DW ± SD] of schisandra lignans in agar microshoot *S. chinensis* cv. Sadova cultures cultured on different MS medium variants after 60 days growth period. Different letters indicate significant differences between means (n = 3, $p<0.05$).

| **Lignans** | **MS medium variants*** | | | | | | |
|---|---|---|---|---|---|---|---|
| | **A** | **B** | **C** | **D** | **E** | **F** | **G** |
| **Schisandrin** | 92.7±2.0 | 46.6±1.6 | 47.7±3.2 | 119.7±6.0 | 75.1±3.5 | 116.5±4.9 | 85.9±4.6 |
| **Gomisin A** | 18.4±0.8 | 8.7±0.1 | 28.4±0.1 | 32.2±0.5 | 27.0±0.3 | 26.8±0.4 | 43.9±0.9 |
| **Angeloyl-/tigloylgomisin H** | 31.5±0.9 | 17.6±2.1 | 30.5±0.9 | 54.6±1.0 | 33.0±0.8 | 38.4±1.1 | 48.8±1.6 |
| **Angeloyl-/tigloylgomisin Q** | 16.7±0.4 | 15.6±0.5 | 45.6±1.5 | 58.9±1.7 | 40.0±1.2 | 29.8±2.5 | 62.2±2.9 |
| **Gomisin G** | 5.4±0.1 | 4.3±0.1 | 10.6±0.3 | 12.9±0.3 | 8.1±0.5 | 7.4±0.5 | 14.4±0.8 |
| **Schisantherin A** | 4.9±0.1 | 1.2±0.1 | 1.8±0.1 | 2.4±0.1 | 3.7±0.3 | 4.6±0.1 | 13.3±2.6 |
| **Schisantherin B** | 22.9±0.3 | 8.1±0.1 | 11.6±0.4 | 21.2±1.7 | 19.3±1.4 | 30.5±1.2 | 44.2±2.6 |
| **Schisanthenol** | 0.6±0.1 | 0.5±0.1 | 1.2±0.1 | 1.4±0.2 | 0.7±0.1 | 0.2±0.1 | 1.2±0.1 |
| **Deoxyschisandrin** | 15.6±0.3 | 12.0±0.1 | 13.4±0.1 | 25.6±1.3 | 15.6±0.7 | 17.8±0.1 | 33.4±1.2 |
| **Schisandrin B** | 6.3±0.1 | 3.1±0.2 | 10.3±0.6 | 19.0±3.4 | 8.6±1.1 | 10.1±0.7 | 12.1±1.8 |
| **γ-Schisandrin** | 4.0±0.2 | 5.3±0.2 | 9.2±0.3 | 22.8±0.7 | 7.3±0.2 | 9.4±0.5 | 16.9±1.3 |
| **Benzoylgomisin P** | 8.0±0.1 | 7.9±0.3 | 16.9±1.1 | 27.9±3.7 | 15.5±0.2 | 16.8±0.7 | 31.4±3.5 |
| **Schisandrin C** | 4.6±0.5 | 2.4±0.2 | 7.9±0.9 | 11.8±1.0 | 7.1±0.2 | 10.9±0.4 | 18.4±1.1 |
| **Schisantherin D** | 19.7±0.5 | 2.9±0.1 | 6.1±0.3 | 13.9±0.8 | 12.9±0.8 | 22.0±2.2 | 37.6±4.7 |
| **Total content** | **251.2±6.1**$^{bcdefg}$ | **136.2±5.6**$^{acdefg}$ | **241.3±9.7**$^{abdefg}$ | **424.2±22.5**$^{abcefg}$ | **274.0±11.3**$^{abcdfg}$ | **341.0±15.3**$^{abcdeg}$ | **463.6±29.7**$^{abcdef}$ |

*A- control (without PGRs), B- 0.1 mg/l BA and 2 mg/l NAA, C- 0,5 mg/l BA and 2 mg/l NAA, D- 2 mg/l BA and 0.5 mg/l NAA, E- 2 mg/l BA and 1 mg/l NAA, F- 2 mg/l BA and 2 mg/l NAA, G- 3 mg/l BA and 1 mg/l NAA

$^{a}$ $p<0.05$ *vs*. MS medium variant A; $^{b}$ $p<0.05$ *vs*. MS medium variant B; $^{c}$ $p<0.05$ *vs*. MS medium variant C; $^{d}$ $p<0.05$ *vs*. MS medium variant D; $^{e}$ $p<0.05$ *vs*. MS medium variant E; $^{f}$ $p<0.05$ *vs*. MS medium variant F; $^{g}$ $p<0.05$ *vs*. MS medium variant G

Table S7. Accumulation [mg/100 g DW ± SD] of schisandra in agitated microshoot *S. chinensis* cv. Sadova cultures cultured on different MS medium variants after 10 days growth period. Different letters indicate significant differences between means (n = 3, $p<0.05$).

| **Lignans** | **MS medium variants*** | |
|---|---|---|
| | **F** | **G** |
| **Schisandrin** | 55.3±1.5 | 69.2±3.5 |
| **Gomisin A** | 30.0±0.7 | 32.0±0.2 |
| **Angeloyl-/tigloylgomisin H** | 21.9±1.1 | 26.9±1.2 |
| **Angeolyl-/tigloylgomisin Q** | 15.5±1.9 | 13.8±1.4 |
| **Gomisin G** | 6.8±0.2 | 7.3±0.3 |
| **Schisantherin A** | 2.9±0.1 | 6.2±0.3 |
| **Schisantherin B** | 15.0±1.6 | 26.2±1.4 |
| **Schisanthenol** | 1.2±0.2 | 1.6±0.1 |
| **Deoxyschisandrin** | 15.1±0.3 | 19.1±0.7 |
| **Schisandrin B** | 4.8±0.1 | 6.5±0.2 |
| **γ-Schisandrin** | 5.1±0.7 | 4.0±0.3 |
| **Benzoylgomisin P** | 8.8±0.8 | 6.8±0.3 |
| **Schisandrin C** | 3.7±0.1 | 3.0±0.3 |
| **Schisantherin D** | 11.2±1.1 | 20.0±0.6 |
| **Total content** | **197.2±10.3**$^{b}$ | **242.8±10.7**$^{a}$ |

* F- 2 mg/l BA and 2 mg/l NAA, G- 3 mg/l BA and 1 mg/l NAA

$^{a}$ $p<0.05$ *vs*. MS medium variant F; $^{b}$ $p<0.05$ *vs*. MS medium variant G

Table S8. Accumulation [mg/100 g DW ± SD] of schisandra lignans in agitated microshoot *S. chinensis* cv. Sadova cultures cultured on different MS medium variants after 20 days growth period. Different letters indicate significant differences between means (n = 3, $p<0.05$).

| **Lignans** | **MS medium variants*** | |
|---|---|---|
| | **F** | **G** |
| **Schisandrin** | 82.9±1.5 | 87.4±1.1 |
| **Gomisin A** | 38.1±0.8 | 41.5±0.9 |
| **Angeloyl-/tigloylgomisin H** | 32.4±0.7 | 33.2±1.3 |
| **Angeloyl-/tigloylgomisin Q** | 31.1±1.0 | 29.2±2.0 |
| **Gomisin G** | 9.1±0.3 | 9.5±0.2 |
| **Schisantherin A** | 6.2±0.4 | 4.1±0.2 |
| **Schisantherin B** | 15.6±0.8 | 19.0±1.1 |
| **Schisanthenol** | 2.3±0.2 | 3.6±0.1 |
| **Deoxyschisandrin** | 24.3±0.3 | 27.3±0.8 |
| **Schisandrin B** | 10.3±0.4 | 0.4±0.1 |
| **γ-Schisandrin** | 9.7±0.7 | 10.5±0.8 |
| **Benzoylgomisin P** | 17.7±0.4 | 14.8±0.2 |
| **Schisandrin C** | 6.1±0.1 | 5.3±0.2 |
| **Schisantherin D** | 16.5±1.0 | 18.5±0.6 |
| **Total content** | **302.2±8.5** | **304.2±9.7** |

*F- 2 mg/l BA and 2 mg/l NAA, G- 3 mg/l BA and 1 mg/l NAA

Table S9. Accumulation [mg/100 g DW ± SD] of schisandra lignans in agitated microshoot *S. chinensis* cv. Sadova cultures cultured on different MS medium variants after 30 days growth period. Different letters indicate significant differences between means (n = 3, $p<0.05$).

| **Lignans** | **MS medium variants*** | |
|---|---|---|
| | **F** | **G** |
| **Schisandrin** | 83.7±5.3 | 112.3±6.8 |
| **Gomisin A** | 58.0±3.2 | 71.4±6.9 |
| **Angeloyl-/tigloylgomisin H** | 35.5±1.2 | 44.8±1.9 |
| **Angeloyl-/tigloylgomisin Q** | 31.2±2.9 | 30.6±0.8 |
| **Gomisin G** | 8.9±0.7 | 6.5±4.3 |
| **Schisantherin A** | 1.4±0.1 | 7.5±0.2 |
| **Schisantherin B** | 9.6±0.4 | 29.9±1.4 |
| **Schisanthenol** | 2.6±0.1 | 8.4±1.9 |
| **Deoxyschisandrin** | 26.1±0.3 | 21.5±0.5 |
| **Schisandrin B** | 11.5±0.6 | 0.4±0.2 |
| **γ-Schisandrin** | 10.4±0.3 | 5.7±0.8 |
| **Benzoylgomisin P** | 22.0±0.6 | 9.0±0.3 |
| **Schisandrin C** | 6.0±0.9 | 4.9±0.2 |
| **Schisantherin D** | 12.5±1.1 | 22.3±0.5 |
| **Total content** | **319.5±17.8[b]** | **375.1±26.6[a]** |

* F- 2 mg/l BA and 2 mg/l NAA, G- 3 mg/l BA and 1 mg/l NAA

[a] $p<0.05$ *vs*. MS medium variant F; [b] $p<0.05$ *vs*. MS medium variant G

Table S10. Accumulation [mg/100 g DW ± SD] of schisandra lignans in agitated microshoot *S. chinensis* cv. Sadova cultures cultured on different MS medium variants after 40 days growth period. Different letters indicate significant differences between means (n = 3, $p<0.05$).

| **Lignans** | **MS medium variants*** | |
|---|---|---|
| | **F** | **G** |
| **Schisandrin** | 56.4±2.0 | 83.1±2.6 |
| **Gomisin A** | 51.3±1.9 | 59.8±3.5 |
| **Angeloyl-/tigloylgomisin H** | 16.9±3.2 | 29.9±1.1 |
| **Angeloyl-/tigloylgomisin Q** | 14.7±0.7 | 16.0±1.2 |
| **Gomisin G** | 3.7±0.1 | 9.29±0.1 |
| **Schisantherin A** | 3.7±0.5 | 7.34±0.2 |
| **Schisantherin B** | 11.8±1.3 | 29.4±0.6 |
| **Schisanthenol** | 2.0±0.2 | 4.7±3.1 |
| **Deoxyschisandrin** | 12.4±0.1 | 18.1±0.5 |
| **Schisandrin B** | 7.5±0.1 | 0.5±0.3 |
| **γ-Schisandrin** | 3.8±0.3 | 2.0±0.1 |
| **Benzoylgomisin P** | 7.8±0.1 | 3.2±0.2 |
| **Schisandrin C** | 1.5±0.1 | 1.9±0.2 |
| **Schisantherin D** | 6.1±0.3 | 16.1±1.5 |
| **Total content** | **199.5±10.7$^{b}$** | **281.5±15.0$^{a}$** |

* F- 2 mg/l BA and 2 mg/l NAA, G- 3 mg/l BA and 1 mg/l NAA

$^{a}$ $p<0.05$ *vs*. MS medium variant F; $^{b}$ $p<0.05$ *vs*. MS medium variant G

Table S11. Accumulation [mg/100 g DW ± SD] of schisandra lignans in agitated microshoot *S. chinensis* cv. Sadova cultures cultured on different MS medium variants after 50 days growth period. Different letters indicate significant differences between means (n = 3, $p<0.05$).

| **Lignans** | **MS medium variants*** | |
|---|---|---|
| | **F** | **G** |
| **Schisandrin** | 47.6±0.9 | 41.6±1.0 |
| **Gomisin A** | 22.4±0.6 | 20.1±1.2 |
| **Angeloyl-/tigloylgomisin H** | 13.1±1.8 | 15.8±0.7 |
| **Angeloyl-/tigloylgomisin Q** | 6.1±0.3 | 13.6±0.7 |
| **Gomisin G** | 1.3±0.1 | 5.4±0.1 |
| **Schisantherin A** | 3.5±0.1 | 4.4±0.1 |
| **Schisantherin B** | 14.7±1.3 | 14.3±0.9 |
| **Schisanthenol** | 1.2±0.2 | 1.5±0.1 |
| **Deoxyschisandrin** | 15.4±0.3 | 12.5±0.1 |
| **Schisandrin B** | 3.7±0.2 | 0.6±0.1 |
| **γ-Schisandrin** | 3.3±0.3 | 2.7±0.1 |
| **Benzoylgomisin P** | 7.1±0.2 | 3.6±0.1 |
| **Schisandrin C** | 2.9±0.2 | 0.5±0.1 |
| **Schisantherin D** | 16.6±0.8 | 2.4±0.2 |
| **Total content** | **158.9±7.1[b]** | **138.8±5.4[a]** |

* F- 2 mg/l BA and 2 mg/l NAA, G- 3 mg/l BA and 1 mg/l NAA

[a] $p<0.05$ *vs*. MS medium variant F; [b] $p<0.05$ *vs*. MS medium variant G

Table S12. Accumulation [mg/100 g DW ± SD] of schisandra lignans in agitated microshoot *S. chinensis* cv. Sadova cultures cultured on different MS medium variants after 60 days growth period. Different letters indicate significant differences between means (n = 3, $p<0.05$).

| **Lignans** | **MS medium variants*** | |
|---|---|---|
| | **F** | **G** |
| **Schisandrin** | 26.8±2.8 | 49.7±2.9 |
| **Gomisin A** | 17.6±0.9 | 33.4±0.4 |
| **Angeloyl-/tigloylgomisin H** | 12.2±1.0 | 11.9±0.3 |
| **Angeloyl-/tigloylgomisin Q** | 7.8±0.3 | 4.0±0.2 |
| **Gomisin G** | 4.7±0.3 | 1.4±0.1 |
| **Schisantherin A** | 3.4±0.1 | 3.2±0.1 |
| **Schisantherin B** | 14.6±1.7 | 16.7±1.9 |
| **Schisanthenol** | 3.3±0.4 | 0.7±0.1 |
| **Deoxyschisandrin** | 14.8±0.3 | 14.9±0.2 |
| **Schisandrin B** | 2.6±0.1 | 0.1±0.1 |
| **γ-Schisandrin** | 1.3±0.1 | 1.5±0.1 |
| **Benzoylgomisin P** | 2.4±0.1 | 2.5±0.5 |
| **Schisandrin C** | 0.6±0.1 | 1.5±0.2 |
| **Schisantherin D** | 8.0±0.6 | 13.8±0.2 |
| **Total content** | **120.0±8.6**$^{b}$ | **155.2±7.2**$^{a}$ |

* F- 2 mg/l BA and 2 mg/l NAA, G- 3 mg/l BA and 1 mg/l NAA

$^{a}$ $p<0.05$ *vs*. MS medium variant F; $^{b}$ $p<0.05$ *vs*. MS medium variant G
